# A velocity-loop view of avian gait: hodograph rotation sense characterizes the walking-to-running continuum in guinea fowl

**DOI:** 10.1242/bio.062880

**Published:** 2026-09-18

**Authors:** Monica A. Daley, Aleksandra Birn-Jeffery

**Affiliations:** ^1^ Department of Ecology and Evolutionary Biology, University of California, Irvine, Irvine, CA 92697, USA; ^2^ Department of Biomedical Engineering, Henry Samueli School of Engineering, University of California, Irvine, Irvine, CA 92697, USA; ^3^ Department of Mechanical and Aerospace Engineering, Henry Samueli School of Engineering, University of California, Irvine, Irvine, CA 92697, USA; ^4^ School of Sport, Rehabilitation and Exercise Sciences, University of Essex, Colchester CO4 3SQ, UK

**Keywords:** Gait transition, Grounded running, Center of mass, Hodograph, Collision, Guinea fowl

## Abstract

Terrestrial gaits are usually treated as discrete categories, yet the criteria that define them disagree in ground birds, which use grounded-running, bouncing dynamics without an aerial phase, over much of their speed range. We asked whether the rotation sense of the center-of-mass velocity loop, the hodograph, resolves gait-transition speeds in guinea fowl (*Numida meleagris*). We reconstructed center-of-mass motion from ground reaction forces over 2580 level overground steps (dimensionless speed range: 0.14 to 3.27). Hodograph rotation sense reverses from pendular to bouncing at a crossover speed of 0.58, close to 0.56 from kinetic-potential energy congruity and well below 1.06 at which aerial phases appear. Six established gait descriptors show the same structure: one principal component explains 50% of their variance and describes a pendular-to-bouncing continuum, and clustering agrees with hodograph rotation sense on 87.7% of steps. Gait in guinea fowl is a continuum whose most meaningful division is walking to grounded running, which can be determined based on one signed scalar per cycle from the velocity trajectory. The approach requires no measurement of height, making it less sensitive to integration drift, which may open opportunities for comparative gait studies in freely ranging animals equipped with inertial sensors.

## Teaser

**Summary:** In guinea fowl, walking and running form a continuum of center-of-mass dynamics in which the rotation sense of the velocity loop indicates the walk-run divide.

## INTRODUCTION

Gaits are classically defined by their footfall patterns (Hildebrand, 1965, 1989), and these kinematic characteristics remain a basis of the walk-run distinction. Bipedal walking typically has overlapping foot contacts and a duty factor above 0.5, whereas aerial running has periods with no foot-ground contact and a duty factor below 0.5 (Alexander, 2004; Gatesy and Biewener, 1991). Cavagna (1975) and Cavagna et al. (1976, 1977) introduced a mechanical basis for the walk-run distinction, based upon two identified energy-fluctuation mechanisms: inverted-pendulum exchange in walking with kinetic energy (KE) and potential energy (PE) out of phase and bouncing elastic energy exchange in running, with PE and KE fluctuating in phase. A bipedal spring-loaded inverted-pendulum template model with a compliant stance leg can reproduce the body center-of-mass (CoM) trajectories and ground-force patterns of both walking and running, such that vaulting and bouncing appear as distinct operating regions of a single dynamical system (Alexander, 1992; Blickhan, 1989; Geyer et al., 2006; McGeer, 1990a,b, 1992; McMahon and Cheng, 1990). In humans, the transition between walking and running is abrupt, and the kinematic and mechanical definitions agree (Cavagna et al., 1976; Hubel and Usherwood, 2013; Kram et al., 1997; Minetti and Alexander, 1997); however, in ground birds, the kinematic and mechanical criteria for gait definitions diverge.

Ground birds use grounded-running, bouncing CoM dynamics with no aerial phase, over much of their speed range, shifting from inverted-pendulum vaulting to bouncing grounded running at intermediate speeds while the duty factor stays above 0.5 (Daley et al., 2016; Gatesy and Biewener, 1991; Hancock et al., 2007; Usherwood et al., 2008). Free-ranging and small birds spend much of their speed range in grounded running (Andrada et al., 2015; Bishop et al., 2018; Daley et al., 2016; Gatesy and Biewener, 1991; Hancock et al., 2007). Assuming dynamic similarity, bipeds are expected to change from a walk to a grounded run near a relative velocity of 0.6 to 0.8 (Alexander, 1977; Gatesy and Biewener, 1991). Across 12 ground-bird species spanning a 1780-fold range in body mass, kinematic and kinetic parameters have been found to change continuously with speed, whereas, in humans, all but one kinematic parameter changed abruptly at the walk-run transition (Bishop et al., 2018). The human walk-run transition is therefore abrupt, while the avian repertoire is comparatively continuous. For ground birds, footfall patterns and duty factors are not sufficient to determine gait, and it remains unclear how many distinct gaits occur.

The CoM velocity hodograph can be used to determine gait from CoM dynamics alone, and its sense of rotation distinguishes pendular from bouncing exchange. Plotting the CoM velocity vector as a closed loop produces the hodograph of classical dynamics (Greenwood, 1988). The use of loop shape and circulation to classify gait was developed by Kuo (2001, 2002, 2007) and Kuo et al. (2005) from the simplest inverted-pendulum walking model and its step-to-step transition, and empirical human-walking loops were measured by Adamczyk and Kuo (2009). Ruina et al. (2005) put forward a collision-based interpretation of gait energetic costs using hodograph analysis. A quantitative collision counterpart, the collision angle, approximates the dimensionless mechanical cost of transport and rises from walking to running (Lee et al., 2011, 2013). Counterclockwise circulation of the hodograph is pendular (out-of-phase KE and PE), and clockwise is bouncing (in-phase KE and PE), so the sign of the hodograph's circulation distinguishes walking from running (Fig. 1).

Here, we test whether the CoM velocity loop (hodograph) distinguishes gaits in guinea fowl. Our general hypothesis, based on the compliant spring-loaded inverted pendulum (SLIP) template of walking and running (Alexander, 1992; Blickhan, 1989; Geyer et al., 2006; McGeer, 1990a,b; McGeer, 1992; McMahon and Cheng, 1990), is that the space of CoM-dynamics descriptors is dominated by a single continuum axis separating pendular from bouncing energy exchange. We test whether the change in hodograph rotation occurs at a consistent and predictable dimensionless speed and whether it consistently distinguishes between pendular- and bouncing-step cycles in guinea fowl. We expect that grounded and aerial running steps will exhibit the same hodograph rotation sense, opposite that of walking, because they both exhibit bouncing in-phase energy fluctuations. Another purpose of this study is to provide an openly available reference dataset for guinea fowl gait-speed dynamics (Dryad, https://doi.org/10.5061/dryad.7m0cfxqc4) over a broad range of level overground locomotion speeds.

## RESULTS

We analyzed the dimensionality of the gait space using two analyses: principal component analysis (PCA) and K-medoids (partitioning around medoids, PAM) clustering. Our dataset comprises 2580 steps from 243 trials of level overground locomotion in guinea fowl, with dimensionless speed spanning from 0.14 to 3.27. The PCA and PAM analyses included six continuous CoM-dynamics descriptors: pendular recovery, KE-PE congruity, mechanical cost of transport, collision angle, touchdown energy and duty factor. We analyze the variance structure of these established gait metrics to provide a point of comparison for the analysis of the hodograph signed area, a quantitative measure of the rotation sense and area enclosed within the velocity loop (see Materials and Methods). The classic gait metrics suggest a gait continuum rather than a fixed set of gait categories (Tables S1 and S3). PCA reveals that the first principal component accounts for 50.0% of the variance and the second for 24.7%, so two components account for 74.7% of the six-dimensional variation (Table S1). PC1 is the pendular-to-bouncing gait continuum, with pendular recovery and duty factor loading positively at +0.40 and +0.47 and with KE-PE congruity, collision angle, touchdown energy and mechanical cost of transport loading negatively at −0.41, −0.41, −0.38 and −0.37, respectively. Low-energy pendular gaits sit at the positive end, high-energy bouncing gaits sit at the negative end, and the position along the axis shifts with speed (Fig. 2B). PC2 is a largely gait-independent axis carrying the collision measures, mechanical cost of transport and collision angle (loading at +0.61 and +0.56) and rises with the magnitude of per-step energy change (Table S1, Fig. S3). Those two measures are linked by an approximation and correlate at 0.93 (Table S2), so PC2 is best read as one collisional axis. No descriptor dominates PC1: the six loadings span 0.374 to 0.467 in magnitude, and the two largest are duty factor and KE-PE congruity. On the plane of those two descriptors and on the plane of the first two components, the steps lie in one speed-ordered cloud (Fig. 2A,B).

**Fig. 1.**
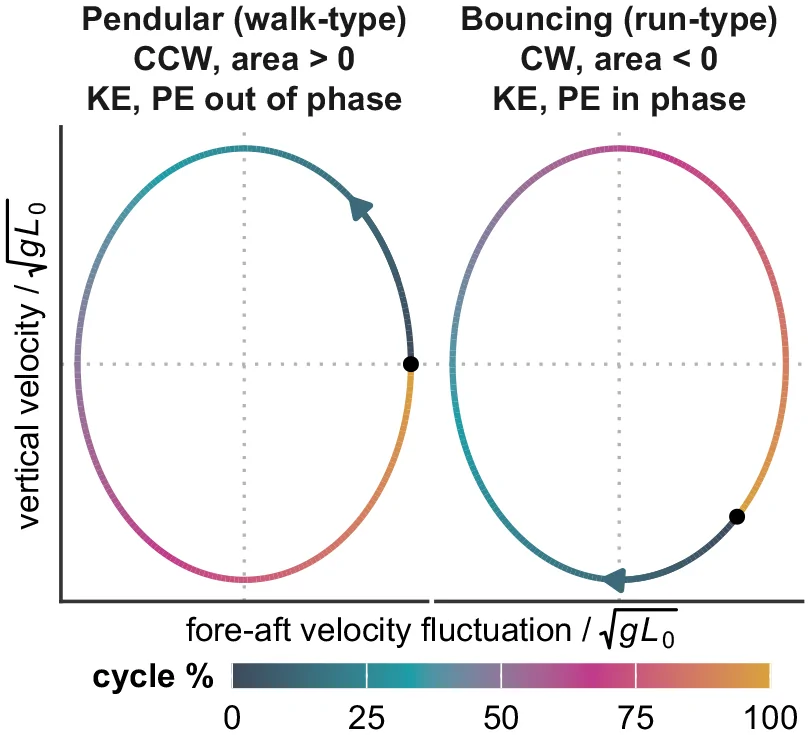
**The sense of rotation of the CoM velocity loop separates pendular from bouncing dynamics.** Two simplified loops in the plane of fore-aft velocity fluctuation (horizontal) and vertical velocity (vertical), both normalized by 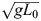. Colors indicate the cycle phase; the arrow indicates the direction of travel, and the dot marks signify the touchdown. A pendular, walk-type step turns counterclockwise (CCW) with a positive enclosed area and KE and PE out of phase; a bouncing, run-type step turns clockwise (CW) with a negative area and the two energies in phase.

**Fig. 2.**
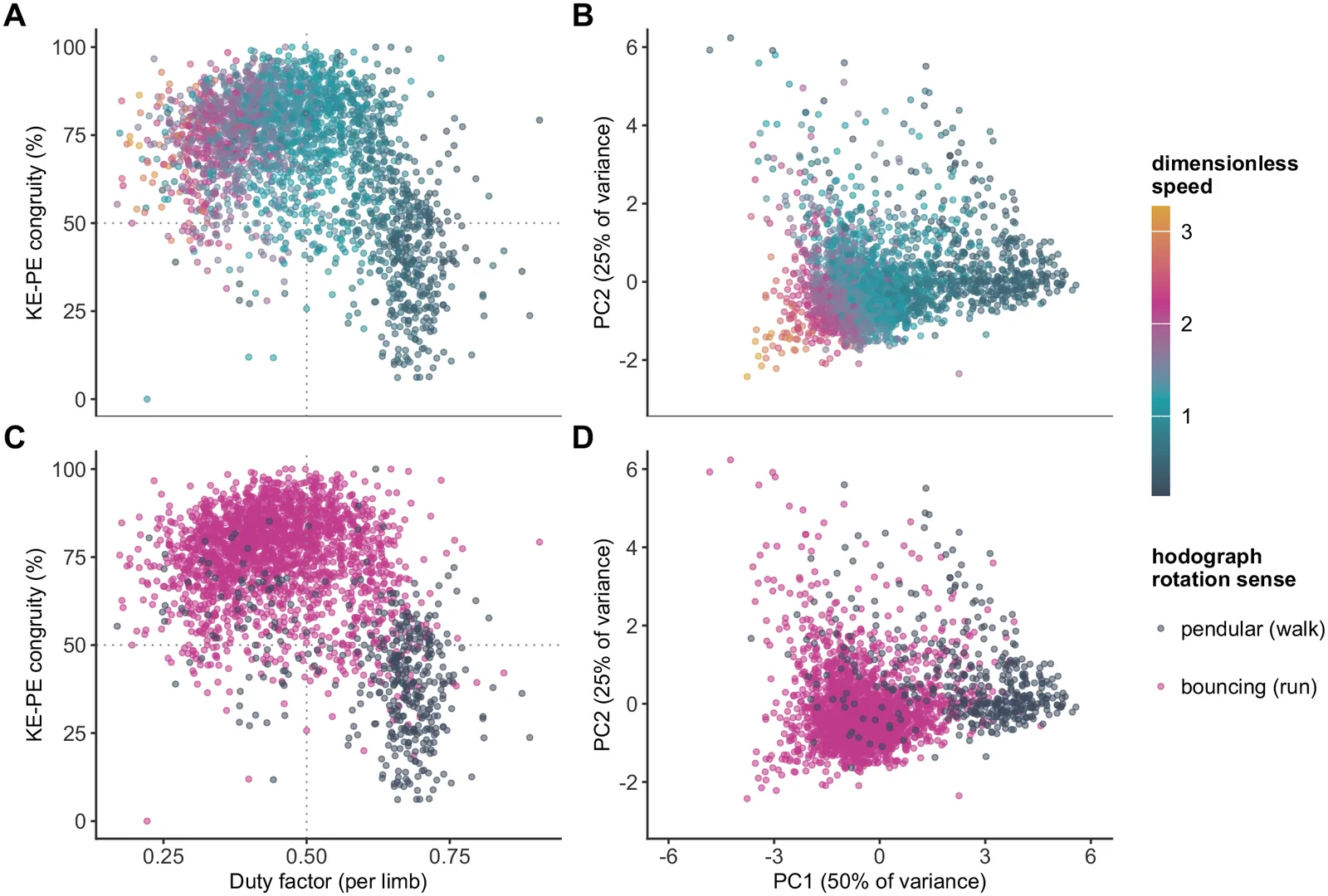
**The CoM-dynamics descriptors form one speed-ordered continuum.** All analysis-sample steps (*n*=2580). (A) The two descriptors loading highest on PC1, duty factor and KE-PE congruity, colored by dimensionless speed; dotted lines mark the duty factor at 0.5 and congruity at 50%. (B) The first two principal components of the six standardized descriptors, colored by dimensionless speed. (C,D) The same two planes colored by hodograph rotation sense, which is not one of the descriptors entering the PCA. Pendular steps are drawn over bouncing steps because they are fewer in number, so the panels show where each class falls rather than how numerous it is. Ten steps fall outside the axis range in B and D.

PAM analysis reveals a weak cluster structure, supporting only a single split, at *k*=2, where the average silhouette width reaches 0.42. The silhouette width falls to 0.27 or below for *k*≥3 (Table S3). The two-cluster split agrees with hodograph rotation sense on 87.7% of steps (2262 of 2580, 95% confidence interval of 84.0 to 91.9 from a cluster bootstrap over the 13 individuals), with a correlation between the two binary labels of *φ*=0.62 and a chance-corrected agreement of *κ*=0.61 (Table S3). Agreement between cluster-based division and hodograph rotation sense is high in aerial running (94.5%, *n*=1617) and lower in the two grounded gaits (80.2%, *n*=329, in walking and 74.1%, *n*=634, in grounded running). The cluster boundary falls at a more bouncing position on the continuum compared to the zero crossing of the hodograph loop area. The two divisions therefore identify the same contrast without coinciding exactly and differ most in intermediate-speed grounded gaits, consistent with a graded boundary. Rotation sense divides both planes along that contrast, with the pendular steps at a high duty factor, low congruity and the positive end of PC1 (Fig. 2C,D). One dominant PC together with one weak clustering split suggests a gait continuum along a single contrast between pendulum-like and bouncing-like mechanics.

Crossing the two classic features of grounded versus aerial and pendular versus bouncing yields four cells, of which three are well populated: grounded and pendular (walk, *n*=329 steps), grounded and bouncing (grounded run, *n*=634) and aerial and bouncing (aerial run, *n*=1544). The fourth cell, aerial and pendular, contains only 73 steps. Assigning gait from the two features is definitional, so the useful information lies in which combinations are used by guinea fowl. The fourth cell is the pendular run: a step with inverted-pendulum stance phases separated by flight phases, which Srinivasan and Ruina (2006) predicted as optimal for a work-minimizing minimal biped at intermediate speeds. However, guinea fowl do not typically use it as a steady gait. Of its 73 steps (3% of all steps), only 8% of these (six steps) are steady, against 36% to 39% steady steps in the three populated gaits. This is most consistent with a transient change in speed and suggests that a flight phase taken from a vaulting stance is a transient behavior rather than a gait typically used by guinea fowl.

Hodograph rotation sense, measured from the CoM velocity loop, describes approximately the same gait space that emerges from the PCA and PAM clustering of traditional gait descriptors. The change in rotation is sharp: the crossover speed, where half of the steps are classified as bouncing, occurs at *u*=0.58 (95% confidence interval 0.46 to 0.63; Froude 0.33), and at the slowest speeds, the loops are almost entirely pendular (Fig. 3B). Over the same steps, the crossover speed for the energy-phase criterion (percent congruity) occurs at *u*=0.56 (0.44 to 0.64), so the 95% confidence intervals for the two crossover speeds overlap. The footfall criterion changes at roughly twice that speed: an aerial phase appears at *u*=1.06 (1.01 to 1.09). The mechanical and footfall transitions therefore bracket the relative velocity of 0.6 to 0.8, at which ground birds have been reported to change gait (Gatesy and Biewener, 1991), and the 0.8 expected for mammals on dynamic-similarity grounds (Alexander, 1977). Of the 1617 steps carrying a flight phase, 96% are clockwise (bouncing, Fig. 3A). The hodograph signed area is well defined despite partial reversals within a loop: the velocity vector turns backward over more than 1% of the turning angle in 68% of steps, and those segments account for a median 5% of that angle, and the loop still completes a full turn in one direction. Rotation sense therefore places aerial running firmly on the bouncing side and accurately locates the walk-run transition on a continuous speed axis.

**Fig. 3.**
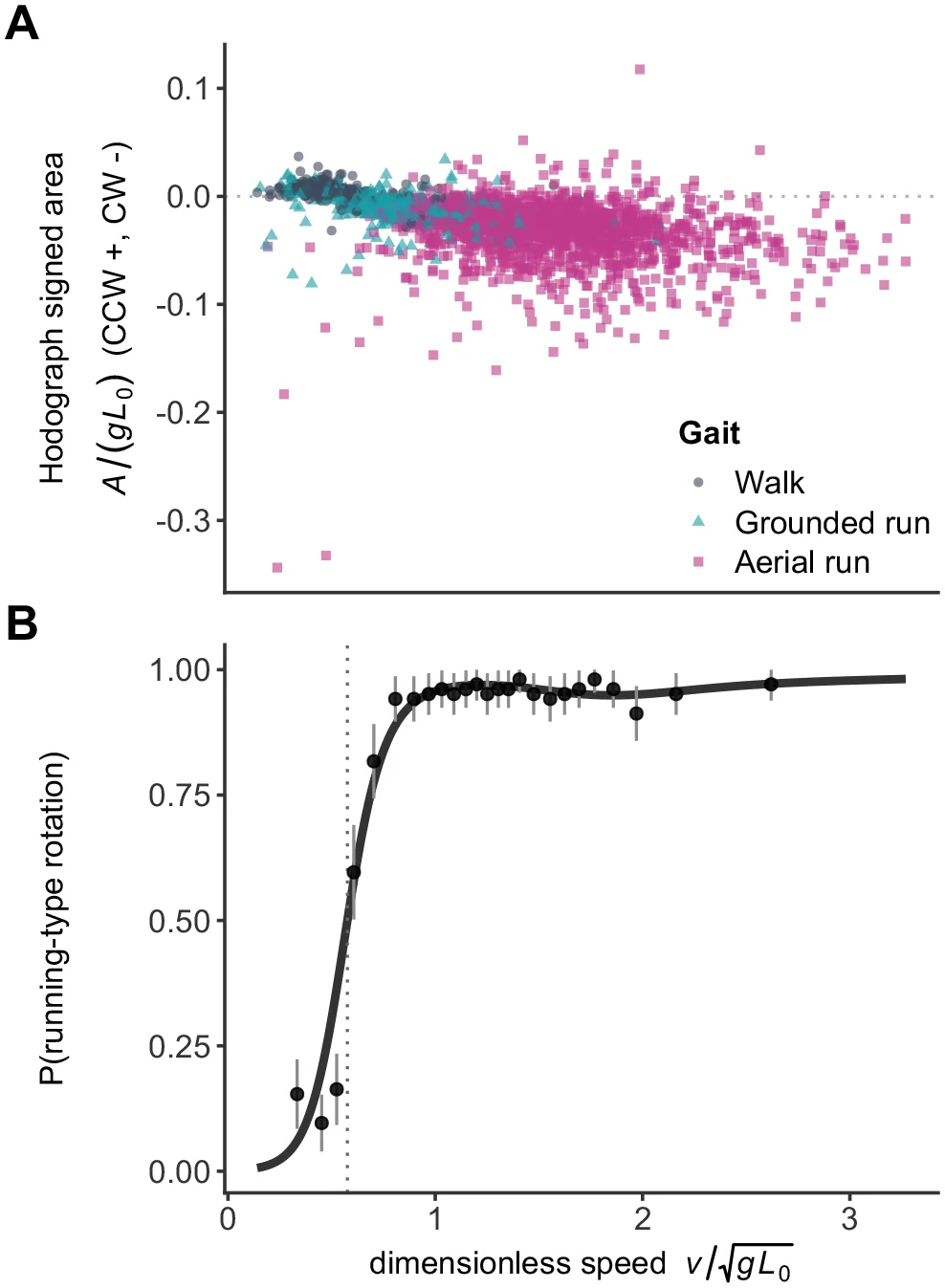
**Hodograph rotation sense reverses sharply with speed near the walk-to-grounded-run boundary.** (A) The signed area of the CoM velocity loop against dimensionless speed, colored by gait. (B) Probability of a clockwise loop against dimensionless speed, with the fitted binomial generalized additive model and empirical proportions in equal-count speed bins. The estimated crossover speed is *u*=0.58.

Established gait descriptors vary in the expected order across the gaits. Across steady steps, pendular recovery falls from a median of 47.9% (interquartile range 35.3 to 66.1) in walking to 5.0% (2.3 to 11.3) in grounded running and 1.6% (0.5 to 4.2) in aerial running. Percent congruity rises from 29.7% (20.3 to 38.5) to 78.4% (68.5 to 84.4) to 81.4% (75.0 to 87.3). Collision angle rises from 6.6° (5.4 to 8.2) to 11.3° (10.2 to 12.6) to 13.0° (11.8 to 14.3), and total CoM mechanical energy at touchdown rises from 0.98 (0.86 to 1.10) to 1.29 (1.13 to 1.45) to 2.06 (1.72 to 2.51) in units of *mgL*_0_ (Table 1). The force- and velocity-weighted collision angle tracks the dimensionless mechanical cost of transport closely but not exactly (Pearson *r*=0.93, *n*=2580 steps). The ratio of the two is centered on 0.99 with an interquartile range of 0.94 to 1.05, and they agree most closely in walking (*r*=0.99) and least in aerial running (*r*=0.90), where the largest collision angles fall outside the range over which the small-angle approximation holds. The magnitude of the hodograph signed area tracks the mechanical cost of transport less closely, at *r*=0.51 over those steps, so the sign of the loop distinguishes the direction of energy exchange while the collision angle quantifies the mechanical work cost. The gait metrics vary continuously with speed, within gaits as much as between them (Fig. 4), and within walking, there is a smooth-to-bouncy gradient in recovery and congruity, so the reduced-recovery walking steps are the bouncy end of walking.

**Fig. 4.**
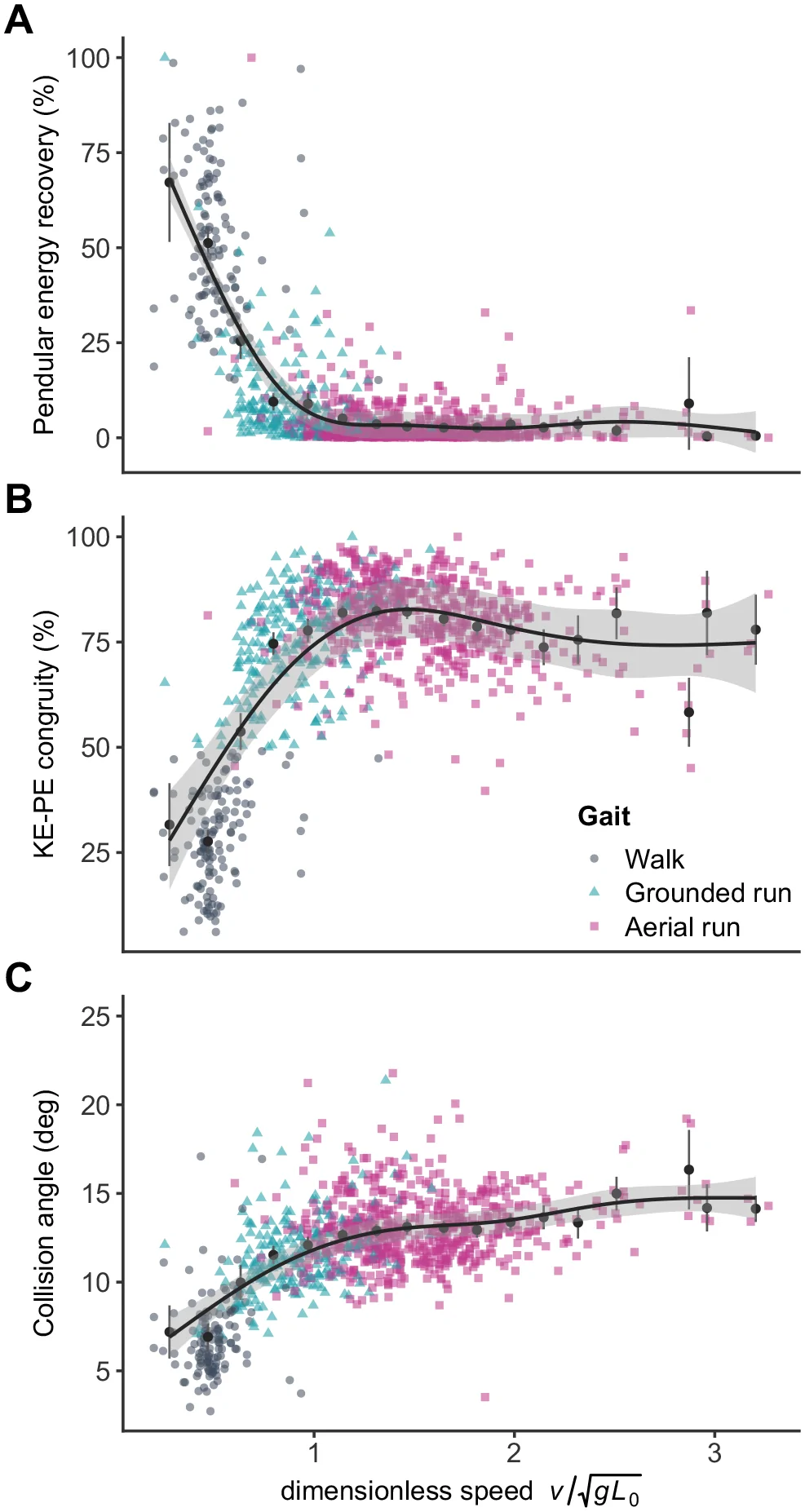
**CoM-dynamics metrics change continuously with speed, within gaits as much as between them.** Steady steps (*n*=930), by gait, against dimensionless speed. (A) Pendular energy recovery. (B) KE-PE congruity, the percentage of the cycle over which KE and PE change in the same direction. (C) Collision angle, in degrees; two steps fall above the axis range. Black points and 95% confidence whiskers indicate speed-bin means, and the curve is a smoothing spline fitted to those means with a 95% confidence band.

**Table 1.** CoM dynamics of steady steps, by gait cell

Measure	Walk (grounded)	Grounded run	Aerial run	Pendular run
Number of steady steps (out of all steps)	127 of 329	229 of 634	568 of 1544	6 of 73
Dimensionless speed, *u*	0.49 (0.44 to 0.56)	0.90 (0.74 to 1.07)	1.49 (1.26 to 1.76)	1.78 (1.46 to 1.91)
Froude number, *u*^2^	0.24 (0.20 to 0.31)	0.82 (0.55 to 1.13)	2.23 (1.59 to 3.10)	3.17 (2.14 to 3.65)
PC1 score	3.82 (3.14 to 4.29)	0.63 (0.10 to 1.13)	−0.78 (−1.24 to −0.34)	0.40 (−0.19 to 1.06)
PC2 score	0.01 (−0.21 to 0.23)	−0.38 (−0.68 to −0.03)	−0.74 (−1.06 to −0.29)	−1.09 (−1.52 to −0.35)
Hodograph rotation sense (% CCW/% CW)	87/13	5/95	1/99	17/83
Hodograph signed area, *A*/(*gL*_0_)	0.0052 (0.0017 to 0.0090)	−0.0116 (−0.0170 to −0.0071)	−0.0243 (−0.0338 to −0.0176)	−0.0121 (−0.0148 to −0.0027)
Pendular recovery (%)	47.9 (35.3 to 66.1)	5.0 (2.3 to 11.3)	1.6 (0.5 to 4.2)	17.9 (10.7 to 29.9)
KE-PE congruity (%)	29.7 (20.3 to 38.5)	78.4 (68.5 to 84.4)	81.4 (75.0 to 87.3)	45.9 (45.2 to 46.9)
Mechanical cost of transport	0.115 (0.096 to 0.142)	0.199 (0.175 to 0.222)	0.227 (0.203 to 0.253)	0.162 (0.066 to 0.201)
Collision angle (deg)	6.6 (5.4 to 8.2)	11.3 (10.2 to 12.6)	13.0 (11.8 to 14.3)	12.7 (9.7 to 15.3)
Touchdown mechanical energy, *E*/(*mgL*_0_)	0.98 (0.86 to 1.10)	1.29 (1.13 to 1.45)	2.06 (1.72 to 2.51)	2.55 (1.88 to 2.87)
Leg stiffness, *kL*_0_/(*mg*)	7.1 (5.0 to 10.2)	6.2 (5.3 to 7.4)	9.7 (7.9 to 12.1)	9.5 (5.4 to 13.3)
Steps with a leg-stiffness fit	109	224	565	6
Duty factor	0.675 (0.652 to 0.704)	0.565 (0.511 to 0.616)	0.410 (0.360 to 0.461)	0.443 (0.329 to 0.461)

The two gait classifier features are crossed to create four possible gait combinations; the unsteady steps of the same cells are shown in Table S4. Values are median (interquartile range).

Leg stiffness is often measured as a parameter for comparing CoM dynamics against simple template models. Over steady steps, dimensionless leg stiffness is 7.1 (5.0 to 10.2) in walking, 6.2 (5.3 to 7.4) in grounded running and 9.7 (7.9 to 12.1) in aerial running, with interquartile ranges that overlap across the three gaits (Table 1).

Phase-averaged hodograph loops for the steady strides of each gait separate walking from both grounded and aerial running by rotation sense (Fig. 5). Ordered by speed, the median signed area falls monotonically across the six cells, from +0.012 in the slow walk to −0.060 in the fast aerial run, so the walking loop contracts as the crossover is approached, and the running loops expand beyond it. Walking traces a small counterclockwise loop in 89% of its steady strides, and that loop is flattest in the fast walk, the cell closest to the crossover speed. Grounded running and aerial running trace larger clockwise loops in 95% and 100% of theirs. These loops differ in size but share the bouncing rotation sense, so grounded running groups with aerial running on this measure. The same progression appears within a single trial: as one bird accelerated from a walk to an aerial run and slowed again, the loops grew and shrank with speed, and only the trial's slowest stride turned counterclockwise (Fig. S4).

**Fig. 5.**
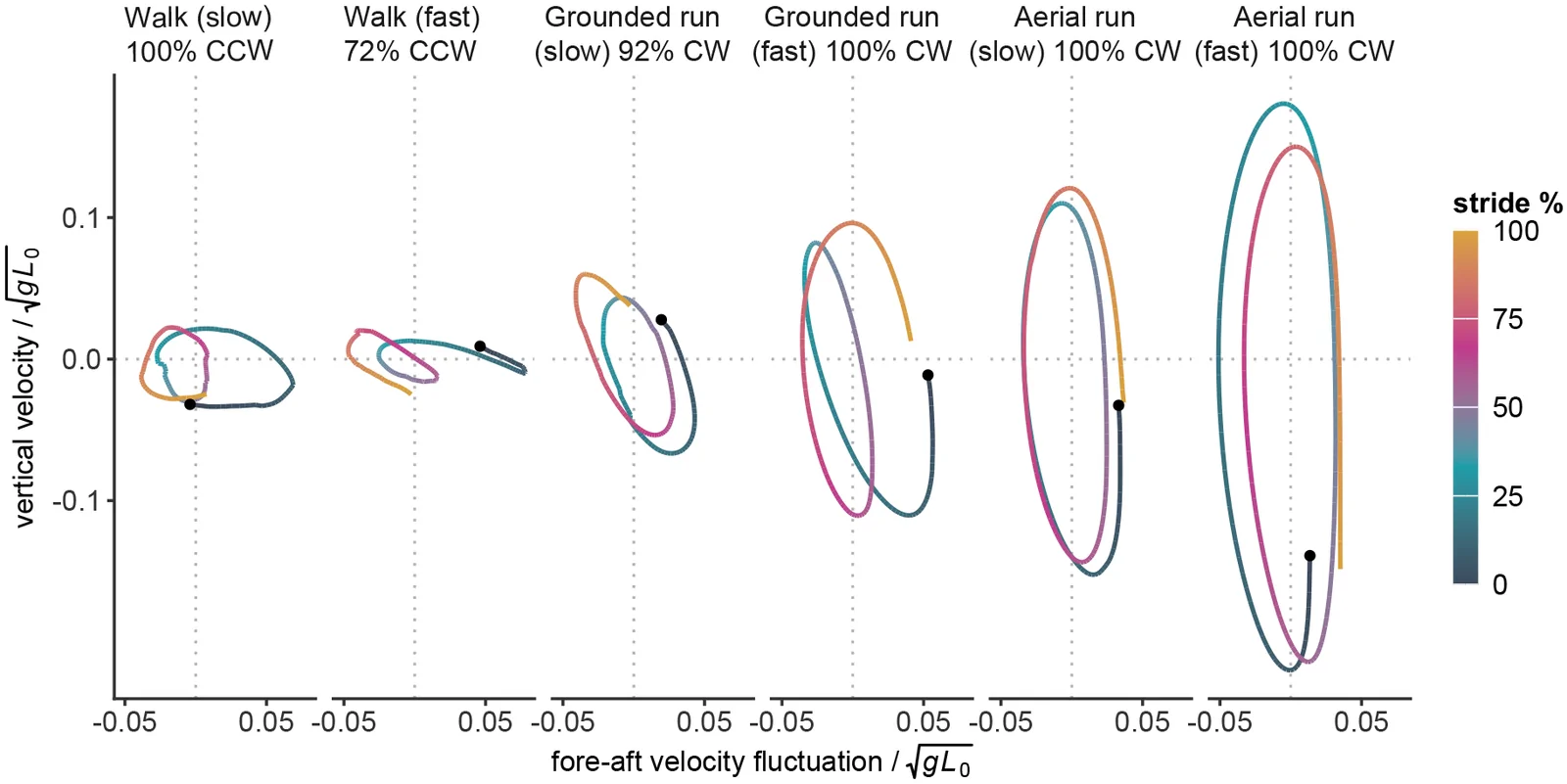
**The hodograph of walking rotates counterclockwise, both grounded and aerial running rotate clockwise, and the enclosed area crosses through zero between them.** Stride-mean hodographs for the steady strides of each gait split slow and fast at the gait's median speed as shown in Fig. 7 and ordered by median speed: fore-aft velocity fluctuation about each stride's mean against vertical velocity, both normalized by 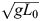. Colors indicate the percentage of stride, and the dot marks indicate the touchdown. The fast-walk loop does not close, so those strides are, on average, slightly decelerating.

Gait-speed characteristics are also consistent with a gait continuum. The duty factor declines with speed and passes 0.5 at the grounded-to-aerial boundary, while step length and step frequency continuously rise with speed without an abrupt transition or gap (Fig. 6A-C). The three gaits occupy overlapping ranges but with distinct preferred speed regions for each gait (Fig. 6D). Over the steady steps, walking spans *u*=0.20 to 1.32 (median 0.49, interquartile range 0.44 to 0.56), grounded running 0.25 to 1.59 (0.90, 0.74 to 1.07) and aerial running 0.47 to 3.27 (1.49, 1.26 to 1.76). The medians separate cleanly, and the distributions are ordered, yet each pair of adjacent gaits shares a wide band of speeds, and grounded running spans most of the walking range. Speed alone therefore does not determine which gait a bird uses.

**Fig. 6.**
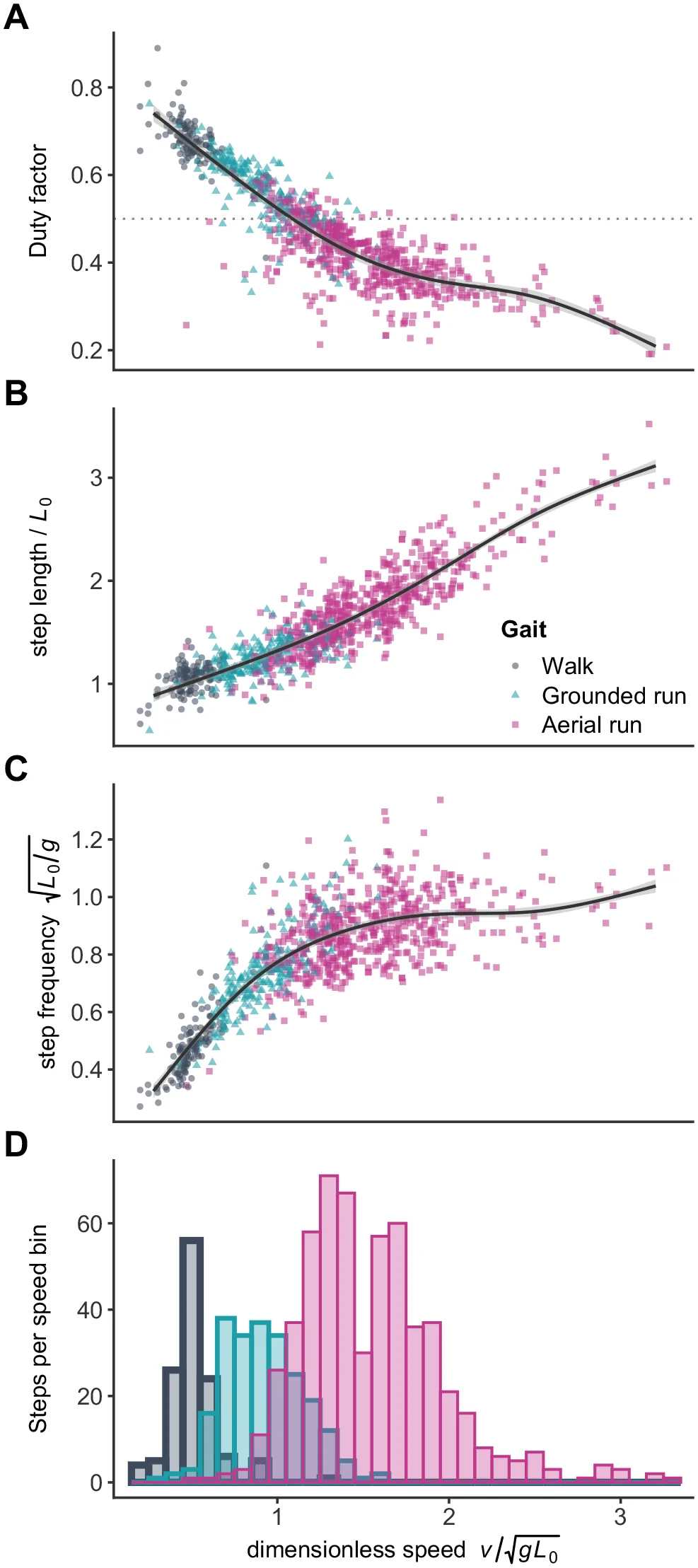
**Speed relations change continuously across the gaits, which overlap in speed.** Steady steps (*n*=930) against dimensionless speed. (A) Duty factor, dotted line at 0.5. (B) Step length normalized by *L*_0_. (C) Step frequency normalized by 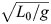. Curves are splines fitted to speed-bin means, with 95% confidence bands. (D) Distribution of the steps over speed by gait.

**Fig. 7.**
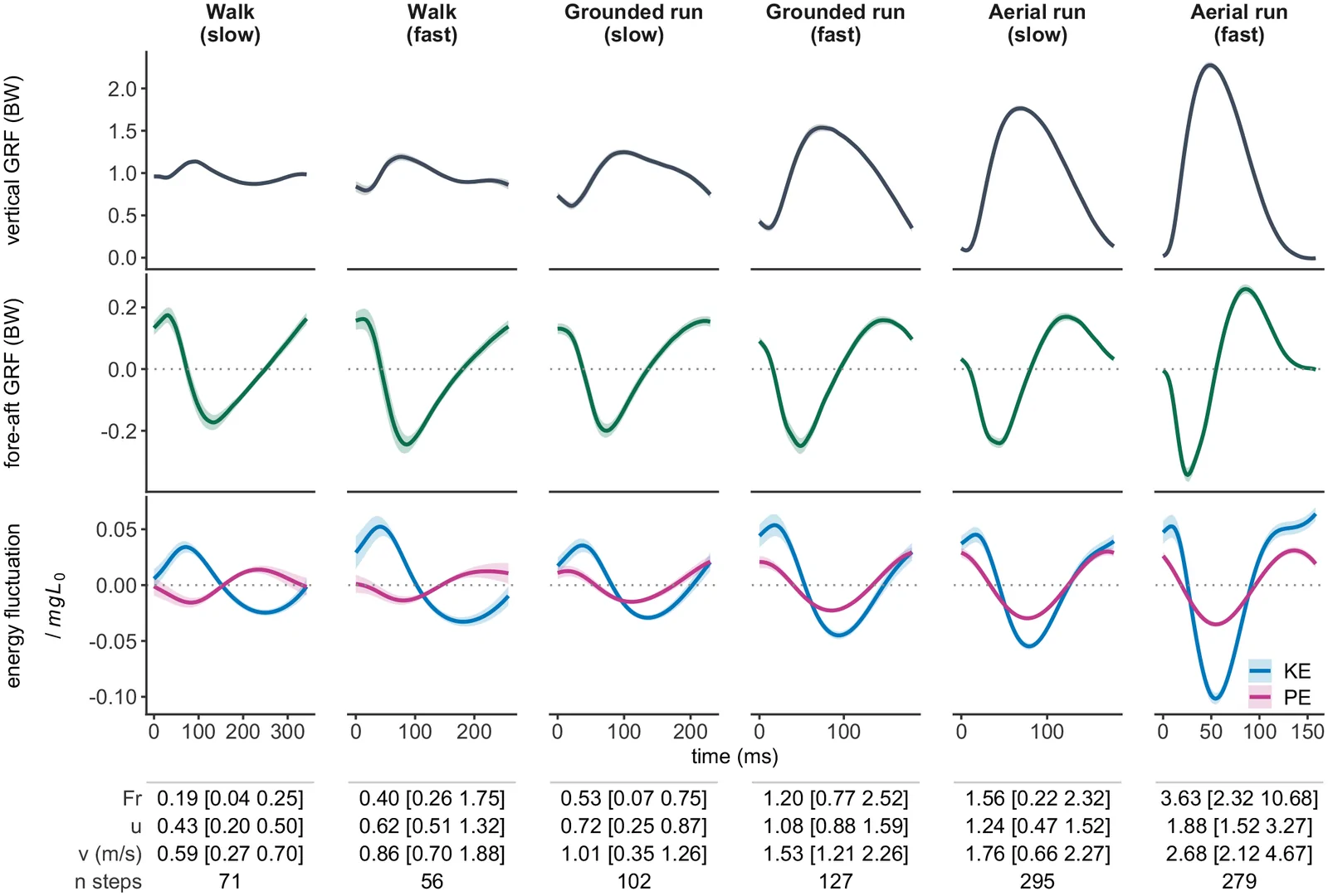
**Force and energy traces shift gradually from pendular to bouncing across gait and speed.** Group mean traces for steady steps with 95% confidence bands; each gait split into slow and fast at its median speed. Rows show the vertical ground reaction force, fore-aft ground reaction force, and CoM KE and PE fluctuation. Inset table at bottom shows the Froude number, dimensionless speed and speed in m/s as mean (minimum and maximum) for each group, along with the number of steps.

Vertical and fore-aft ground reaction force and CoM KE and PE fluctuations, averaged by gait and speed, show the gradual shift in dynamics described by the scalar gait metrics (Fig. 7). The walk traces, split into slow and fast groupings, show the smooth-to-bouncy gradient: vertical force is nearly constant in the slow walk, with the energy traces out of phase, whereas, in the fast walk, a vertical-force peak becomes more prominent and the energy traces shift closer in phase. The run traces show a bounciness gradient, with the vertical-force peak rising and energy traces increasingly in phase as the magnitude of energy fluctuations increases. In every gait, the mean vertical force peaks in the first half of contact, between 27% and 43% of stance across the six groups.

Of the 2580 steps, 930 (36.0%) met the steadiness criterion, 947 were accelerating and 703 were decelerating. The steady subset spans *u*=0.20 to 3.27 against 0.14 to 3.27 for all steps, at a median dimensionless speed of 1.24 against 1.25. The fraction of strides excluded as unsteady was between 55% and 70% across six equal-count speed bins (Fig. S2). Acceleration state alters the traces but occurs in all three gaits (Figs S5 and S6). In the CoM-dynamics principal-component space, the steadiness classes order along the second axis: within every gait, the decelerating centroid sits highest on PC2 and the steady centroid lowest, with the accelerating centroid displaced from the decelerating one along PC1 (Fig. S3). The clouds overlap, so this is an ordering of centroids rather than a separation of steps. The supplement provides a visualization of the steadiness criterion and analysis of the unsteady data.

## DISCUSSION

We asked whether the CoM dynamics of guinea fowl locomotion divide into distinct gaits or exhibit a gait continuum. Six established gait descriptors reduce to a dominant principal component that explains 50.0% of the variance, characterizing a pendular-to-bouncing continuum, and PAM supports a single weak two-cluster split. A second component, explaining 24.7% of the variance, relates to the mechanical cost of transport and rises with the magnitude of per-step energy change. The variance structure of steady gaits is therefore consistent with a continuum along one mechanical contrast that corresponds to the compliant template in which walking and running are operating regions of one dynamical system (Blickhan, 1989; Geyer et al., 2006; McMahon and Cheng, 1990). These findings are also consistent with the continuous change in kinematic and kinetic parameters with speed measured across 12 ground-bird species (Bishop et al., 2018).

The rotation sense of the hodograph velocity loop characterizes the same mechanical contrast. The unsupervised division of the descriptor space agrees with hodograph rotation sense on 87.7% of steps (95% confidence interval 84.0 to 91.9), with a chance-corrected agreement of *κ*=0.61 and 96% of the 1617 steps carrying a flight-phase turn clockwise. Grounded running therefore groups with aerial running on this measure, where a footfall or duty-factor label groups it with walking, consistent with the subaerial mechanical transition reported for ground birds (Andrada et al., 2015; Bishop et al., 2018; Hancock et al., 2007). The most mechanically meaningful divide in avian gait mechanics is between walking and grounded running, not between grounded and aerial running.

Pendular and bouncing exchange coexist within single steps, which is what a gait continuum implies at the scale of one cycle. The CoM velocity trajectory crosses itself in 25% of steps (650 of 2580), forming two lobes of opposite rotation sense, and the crossover fraction is similar in walking, grounded running and aerial running (28%, 28% and 24%, respectively). Because the signed area is the algebraic sum of the lobe areas, a step whose trajectory crosses still has one net sense. Mixed exchange within a cycle is therefore common in guinea fowl, and the sign of the loop area separates the cycles into net pendular and net bouncing without a third category.

Because gait dynamics of ground birds vary gradually and continuously across the speed range (Bishop et al., 2018; Daley and Birn-Jeffery, 2018; Gatesy and Biewener, 1991; Hancock et al., 2007; Usherwood et al., 2008), the detected transition speed depends on how the transition is defined, on the metric used, and on how densely the speed range is sampled. We found the crossover speed for the transition between pendular and bouncing gaits in guinea fowl to occur at *u*=0.58 using hodograph rotation sense and at *u*=0.56 using the energy-phase criterion, with overlapping intervals. Both fall within the gait-transition speed of 0.50 to 0.59 reported for ostriches from a large overground dataset, using a displacement-amplitude criterion that agrees with the energy phase in 84% of walking and 90% of running steps (Daley et al., 2016), in a bird that is 80-fold larger. Both point estimates sit slightly below the relative velocity band of 0.6 to 0.8 reported for birds (Gatesy and Biewener, 1991). However, Gatesy and Biewener (1991) identified 0.6 to 0.8 kinematically, from a 57% decrease in the slope of relative stride frequency within that band, while acknowledging that detecting the transition requires measures of CoM energy fluctuation. Hancock et al. (2007) reported a transition speed range for tinamou from CoM energy fluctuations at Froude 0.4 to 0.6, or *u*=0.63 to 0.77, while acknowledging limited sampling of the slower speed range. The speed ranges of vaulting and bouncing gaits overlap strongly in ground birds (here: Fig. 6D, tinamou: Hancock et al., 2007), so the detected transition speed is sensitive to sparse sampling. In guinea fowl, the two energy-fluctuation criteria identify a transition at *u*=0.58 and *u*=0.56, with an aerial phase appearing at *u*=1.06, so the two criteria bracket the classical transition speed range. Gatesy and Biewener (1991) found no aerial phase below a relative speed of 1.0 in any of the avian species they measured. Our study is limited to a single species but provides more comprehensive sampling across the speed range than is available for most ground birds. Our finding of a gait continuum, as well as our detected transition to aerial running near *u*=1, is consistent with the findings of Bishop et al. (2018) across 12 ground-bird species that included guinea fowl. It will be interesting in future work to investigate whether the hodograph-based crossover speed is consistent across ground-bird species or varies with body size and limb posture, which influence gait-speed mechanics through trade-offs among the demands for body-weight support, economy and stability (Bishop et al., 2018; Daley and Birn-Jeffery, 2018).

Guinea fowl exhibit a consistent asymmetry in the vertical ground reaction force that is present across the gait-speed continuum (Fig. 7). Our mean traces show the same early-peaking vertical force in walking, grounded running and aerial running. A conservative spring-mass leg cannot produce an asymmetric force profile, whereas adding damping reproduces it (Birn-Jeffery et al., 2014). Clemente et al. (2018) also documented this asymmetry across obligately and facultatively bipedal species and suggested that it may relate to a CoM situated forward of the hips. These two potential contributors to asymmetric vertical force, CoM position and leg damping, are not mutually exclusive, and both may contribute to varying degrees across species. It would be interesting for future modeling work to vary these features systematically, to map how posture, limb inertia and damping influence the ground reaction force profile and the gait-speed continuum in bipeds.

As a secondary analysis, we explored estimates of leg stiffness across the gait-speed continuum in guinea fowl. Median dimensionless leg stiffness is 7.1 in walking, 6.2 in grounded running and 9.7 in aerial running, with overlapping interquartile ranges (Table 1). These sit below the 11.9 (mean, s.d. 3.8) previously reported for guinea fowl level running (Blum et al., 2014), which drew on a subset of the current data and inverted leg compression by a different convention, and below the 15 fitted for this species by work-optimal trajectory optimization of a damped compliant leg, which was one of a nonunique set of parameter combinations that could fit the observed data (Birn-Jeffery et al., 2014), so the space of feasible leg stiffness values is broad. Leg stiffness values also vary depending on the assumptions of the calculation and how well the data fit the assumptions (Blum et al., 2009); so estimates are best compared against others made using the same convention. More detailed kinematics would be required to investigate how leg posture, landing geometry, leg stiffness and mechanical energy state influence variation along the gait-speed continuum in guinea fowl.

Hodograph rotation sense and signed area provide another footfall-free analysis of gait dynamics, one that can complement established gait measures in determining how animals shift between pendular and bouncing dynamics. Like other measures based on CoM dynamics, it requires no information about contact timing or footfalls, so it can be applied to CoM fluctuations derived from either force plates or accelerometry. Pendular energy recovery and KE-PE congruity likewise require only CoM-based measures, but as they are conventionally computed, both take gravitational PE from vertical height, which needs a second integration of acceleration. Rotation sense is determined from the signed velocity-loop area, and this is invariant to translation of the trajectory, so a constant error in the vertical-velocity integration does not change it. It may therefore be less sensitive to integration drift compared to height-based measures, which absorb the integration error as a drifting PE term. Hodograph-based analysis of gait opens opportunities for comparative studies over a broad range of species, including measurements from freely ranging animals equipped with inertial measurement units (Daley et al., 2016; Dewhirst et al., 2017; Shepard et al., 2008), which could allow systematic testing of how gait-speed dynamics vary with body size, limb posture and other morphological and ecological factors.

### Conclusion

The hodograph rotation sense and signed area provide a method to classify gait quantitatively from CoM velocity, enabling identification of the pendular-to-bouncing transition in animals that spend much of their speed range in grounded locomotion. In guinea fowl, gait varies gradually across the pendular-to-bouncing continuum, with a crossover speed near a dimensionless speed of 0.6. The rotation sense of the velocity loop marks the division along that axis from the velocity trajectory alone, and it places grounded running and aerial running within the same mechanical regime.

## MATERIALS AND METHODS

### Animals and data collection

We analyzed level overground running trials of guinea fowl (*Numida meleagris*) compiled from three data collections at the Royal Veterinary College: an obstacle, drop-perturbation and level-running collection; a surface-variation study; and a drop-versus-pothole study. We included all level, unperturbed trials in this analysis. The dataset includes 30 bird sessions across 13 individuals, several birds having been recorded in more than one session. Body mass and leg length for the reconstruction were measured per session because a bird's weight varied between sessions. Across the collections, the per-session mass used for the reconstruction and for normalization ranged from 1.09 to 1.93 kg (mean 1.36 kg), and leg length *L*_0_ from 0.192 to 0.232 m (Table S5). The birds were housed and trained as described previously (Birn-Jeffery and Daley, 2012; Birn-Jeffery et al., 2014). The Royal Veterinary College Ethics and Welfare Committee approved all procedures under the project protocol ‘Kinematics and kinetics in birds running over uneven terrain’. The protocols required no invasive or surgical procedures. Ground reaction force was recorded with Kistler force plates (model 9287B, 0.6 m by 0.9 m; Kistler Instruments, Winterthur, Switzerland), with five or six active plates embedded in the runway, recording at 500 Hz and summed across plates to give the net ground reaction force. Marker kinematics were recorded with a Qualisys motion-capture system (Qualisys AB, Gothenburg, Sweden) at 250 Hz. The kinematic estimate of the body COM position is taken from the average of two markers placed in cranial and caudal positions on the bird's back, and the foot position is taken from the average of two markers placed on the tarsometatarsophalangeal joint and the distal phalanx of the 3rd digit, as described previously (Birn-Jeffery et al., 2014). Note that the kinematic CoM estimate was used only for estimating initial conditions for the force-plate-derived body CoM trajectories, as described in the ‘CoM reconstruction’ section. The net force was low-pass filtered before analysis (zero-phase Butterworth, 50 Hz). Foot-marker tracks were cleaned before use: samples with a foot height above 0.4 m, or more than six robust standard deviations from that foot's median height, were removed from all three axes together because an untracked marker extrapolates in every axis at the same samples. The gaps were then filled using shape-preserving interpolation, after which the tracks were low-pass filtered at 20 Hz.

### The analysis sample

From 323 level trials, we detected 3842 steps (1678 strides). We then used a number of quality-control criteria to clean the data for analysis. We used a robust step-frequency and step-length filter (log-MAD, *k*=3.0, applied within dimensionless-speed neighborhoods), combined with a speed-conditional filter that flags merged (half-frequency) and split (double-frequency) miscut cycles. This removed 584 steps (205 strides). The speed-conditional filter uses the identity that forward speed is step length times step frequency: a missed or spurious touchdown moves the frequency away from the speed local median while leaving the speed unchanged (Fig. S1). We also removed steps with a work-energy identity residual above 0.20 or a vertical CoM drift above a duration-normalized bound, the larger of 45 mm and a fixed per-second rate times the trial duration. The bound scales with trial duration, equaling 45 mm at the median-duration trial. We also excluded steps with poor foot-marker quality, which leaves the duty factor undefined, and steps in which the reconstructed CoM height above the contacting foot fell outside the range of 0.4 to 1.6 *L*_0_, which follows from poor marker tracking. After these quality-control steps, 2580 steps and 944 strides remained in the sample. Leg stiffness is not in the quality-control gate because its missing values are failures of the spring-mass fit but are not central to our primary hypothesis. The counts for leg stiffness are given in the supplementary tables where leg stiffness is reported.

We assigned each step and stride a steadiness label from the net change in total CoM mechanical energy per unit distance, expressed as a fraction of body weight. A stride is steady when |Δ*E*_CoM_|≤0.05 *mgL*, with *L* the CoM distance over the stride, which is an effective 5% fore-aft grade (about 2.9°). The label is evaluated per stride, because its touchdown-cut-phase component cancels over a stride, and propagated to the stride's two steps. The ratio is the mean net fore-aft force on the CoM as a fraction of body weight, and *L* grows with speed in step with the absolute energy change, so the criterion is speed and size neutral by construction. Steadiness criteria differ between studies. The exclusion rate this one produces is between 55% and 61% up to *u*=1.6 and rises to 70% in the fastest bin (Fig. S2), so the steady subset thins at the top of the speed range. Steps meeting the criterion were used for the steady-state analyses, and the accelerating and decelerating steps were labeled and analyzed separately.

### CoM reconstruction

The CoM was reconstructed by path-matched double integration of the net ground reaction force over the whole on-plate bout. CoM acceleration is *a*=*F*/*m*+*g*, integrated twice, with one set of integration constants per trial per axis. Force-platform reconstruction of the CoM was introduced for steady gait, where the mean acceleration over a stride is zero (Cavagna, 1975). The path-matching method used here fixes the integration constants per axis by minimizing drift against the marker-based kinematic CoM path, so step-by-step unsteady dynamics can be resolved while the growth of integration drift over time stays bounded (Daley et al., 2006; Goldsmith et al., 2026). Reconstruction accuracy is the per-stride root-mean-square deviation between the force-derived and kinematic vertical CoM position, averaged over the 1129 strides of each gait that pass the force-based quality-control gate, which is the grain at which the reconstruction is a property of the measurement rather than of the analyzed subset: 35 mm in walking (*n*=152 strides), 18 mm in grounded running (*n*=189) and 8 mm in aerial running (*n*=788).

The midpoint of the two back markers lies above and behind the whole-body CoM. We applied a fore-aft and a vertical offset to it before taking any leg geometry so that leg length, leg angle and height above the foot all refer to one CoM. The fore-aft offset was fitted by driving the net pitch impulse about the CoM to zero over stride-complete windows, within anatomical bounds set by the two back markers. That condition does not identify the vertical offset because the fore-aft impulse of the summed ground reaction force approaches zero over a whole stride, so the vertical offset was set from anatomical scaling as −0.2625 *L*_iso_, with *L*_iso_=0.20 *m*^1/3^. Offsets were fitted for each bird in each recording session because marker placement was renewed at each session. The resulting leg angle at touchdown is 122.9° (s.d. 8.9) over the analyzed steps, within the 122.6° (5.4) reported for guinea fowl level running (Blum et al., 2014). Steps and strides were cut at foot-marker touchdowns, and a stride comprises two consecutive steps. We derived the duty factor from foot-marker contact. Travel direction varied by trial and was signed per crossing.

### Normalization

All quantities are dimensionless under dynamic similarity, with a dimensionless speed of 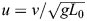 and Froude number of *u*^2^. We normalize by body mass and leg length *L*_0_, each taken for a bird within a recording session, following established dynamic-similarity scaling conventions (Alexander and Jayes, 1983; Daley and Birn-Jeffery, 2018; McMahon and Cheng, 1990). Body mass is the median over the session's trials of the mean vertical ground reaction force through a crossing, divided by *g*. *L*_0_ is the median touchdown length of the virtual leg, from the CoM to the contacting foot, over the session's steps that have no aerial phase. Where a session yielded fewer than five such steps, *L*_0_ was taken from that bird's non-aerial steps pooled over its sessions, and for the one bird that never yielded five, it was from a within-sample allometry of the measured *L*_0_ on body mass. Table S5 gives *L*_0_ and its source for every session. Speed is reported as *u* rather than as a Froude number because the gait measures here change more nearly linearly with *u*, and both are given in Table 1.

### Gait classification from two qualitative features

We classified gait with two binary mechanical features, so the number of gaits follows from which combinations are populated. The first feature is the aerial phase: the summed vertical ground reaction force reaches zero, so there is no ground contact. The second feature is the direction of energy exchange, taken as the classic mechanical walk-run criterion of Cavagna (1975) and Cavagna et al. (1976, 1977) in its per-cycle form. CoM KE and gravitational PE fluctuate out of phase in walking and in phase in running, so we use KE-PE congruity: the percentage of the cycle over which the horizontal KE, 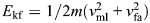, and the vertical energy, 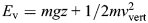, change in the same direction (Ahn et al., 2004). We split it at 50%: out of phase over most of the cycle is pendular, and in phase over most of it is bouncing. Congruity exceeds 50% when the cycle mean of sign(Δ*E*_kf_) sign(Δ*E*_v_) is positive, so the cut is a zero crossing of that mean. Steps falling exactly at 50% are classified as bouncing. The potential gaits are grounded and pendular (walking), grounded and bouncing (grounded running), aerial and bouncing (aerial running), and a fourth combination, aerial and pendular, which is the pendular run predicted by Srinivasan and Ruina (2006). The figures use the three-gait form, in which every step with a flight phase counts as aerial running; however, the four-gait split is included in the summary tables so that the rare fourth gait (pendular run) can be quantified.

### Hodograph rotation sense

Hodograph rotation sense is the sign of the signed area *A* of the CoM velocity loop (Fig. 1), measured from the velocity trajectory alone. For a cycle sampled at *N* points, we set 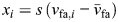, the fore-aft velocity fluctuation about the cycle mean, with *s*=± 1 as the sign of net travel so the measure is travel direction invariant and *y*_*i*_=*v*_vert,*i*_ as the vertical velocity. The signed area follows from the shoelace formula:

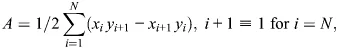
and is reported dimensionless as 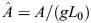, both velocity components having been normalized by 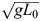. Here, *A*>0 is counterclockwise, indicating pendular (walking-type, out-of-phase KE and PE) dynamics, and *A*<0 is clockwise, indicating bouncing (running-type, in-phase) dynamics, with the boundary at *A*=0. The loop and its collision interpretation follow the Kuo and Ruina lineage (Adamczyk and Kuo, 2009; Kuo, 2001, 2002; Kuo et al., 2005; Ruina et al., 2005). Loops that reverse partway round are classified by their net circulation: where the loop self-intersects, the signed area is the algebraic sum of the lobes, so its sign follows the sense that dominates by area. We quantify how often a reversal occurs as the fraction of the total turning angle of the velocity vector that runs against the net sense. We also count the crossovers of each cycle, taken over the segments joining consecutive samples so that a crossover is a property of the trajectory traversed.

We report the counterclockwise fraction of rotation sense within each gait cell, and the crossover speed of rotation sense, of the energy-phase criterion and of the aerial phase, all fitted over the same steps. The aerial phase is taken from the summed vertical force rather than from the duty factor at 0.5, where the same steps occur at the same speed, because it does not depend on foot-marker contact. The relationship between the loop area and the mechanical cost of transport is quantified as the Pearson correlation between |*A*|/(*gL*_0_) and the dimensionless cost of transport, overall and within each gait.

### Continuous CoM descriptors

We computed six established descriptors as continuous measures mapped onto the gaits. Pendular energy recovery follows Cavagna et al. (1976, 1977). KE-PE congruity has been defined above (Ahn et al., 2004). The collision angle is Φ=arcsin(|*F* · *V*|/(|*F*| |*V*|)), from which we take its force- and velocity-weighted mean, the collision fraction and the dimensionless mechanical cost of transport; the weighted Φ approximates the mechanical cost of transport (Lee et al., 2011, 2013). That link is an approximation in two stages: the small-angle substitution sinΦ≈Φ, which holds below about 0.3 rad, and the assumption that the mean magnitudes of the velocity and force vectors are close to the mean forward speed and to body weight. Together, they give 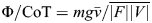, so the two coincide exactly when the mean force-velocity product equals body weight times mean forward speed and departs as it varies. The approximation is stated for Φ in radians, which is the form the correlation uses; the collision angle is reported in degrees in Table 1 and in the text, for readability. The duty factor is the continuous form of the classic walk-run criterion.

Total CoM mechanical energy at touchdown is reported as a dimensionless quantity, calculated as 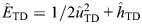, with 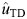 being the sagittal CoM velocity magnitude at touchdown normalized by 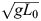 and 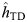 being the CoM height above the contacting foot in units of *L*_0_, taken from the virtual leg as *L*_TD_sin*α*_TD_. Steps whose reconstructed touchdown height is implausible are excluded at the gate (see ‘The analysis sample’ section).

Dimensionless leg stiffness is 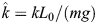, in body weights per leg length (Geyer et al., 2006), from *k*=*F*_max_/Δ*L*, with *F*_max_ being the peak vertical force and the leg compression Δ*L* recovered by inverting a sinusoidal vertical force profile over contact (Blum et al., 2009; Morin et al., 2005):

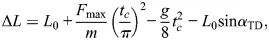
where *t*_*c*_ is the per-limb contact time and *α*_TD_ is the leg angle from horizontal at touchdown. Blum et al. (2009) compared five leg-compression conventions against bipedal spring-mass predictions and found this one to be accurate where the symmetric-trajectory convention underestimates stiffness (Farley et al., 1993; McMahon and Cheng, 1990). Steps returning a nonphysical compression (Δ*L*≤0, or above 0.4*L*_0_) were excluded. A leg that extends over stance returns Δ*L*≤0, which happens most often in walking, so the fraction of steps carrying a stiffness estimate is lowest in that gait. Leg stiffness is reported as a model-bound descriptor. It exists only relative to the spring-mass template from which it is derived (Blum et al., 2009), and where both limbs share the net force in double support, it is an effective two-limb stiffness rather than a single-limb property (Farley et al., 1993; Geyer et al., 2006; McMahon, 1985).

### Statistical and data analysis

The six established continuous gait descriptors defined above were z-scored across the analysis sample. We applied PCA to the standardized matrix and report the loadings and the variance accounted for by the leading components, orienting PC1 with the pendular end positive and PC2 with the unsteady end positive. How far these descriptors reduce to fewer axes depends on how far they are already redundant, so we report their correlation matrix alongside the components, with the hodograph signed area appended to it (Table S2). For cluster tendency, we partitioned the steps around medoids for *k*=2 to 6 and scored each partition by its average silhouette width (Kaufman and Rousseeuw, 1990; Rousseeuw, 1987). We then asked how far the best-supported partition agrees with hodograph rotation sense. We aligned the cluster with the higher proportion of counterclockwise steps with the pendular sense and summarized the resulting two-by-two table with the three agreement statistics described below, overall and within each of the three populated gaits.

One statistical model is fitted. How a binary criterion changes with speed is fitted as a binomial generalized additive model, *P*(bouncing)∼*s*(*u*)+*s*(individual), with a per-individual random-effect smooth over the 13 individuals. The fit is a penalized spline, which allows the data set to determine the sharpness of the change rather than imposing a fixed sigmoid shape. From it, we take the crossover speed, the dimensionless speed at which the fitted population probability reaches 0.5, so that a step drawn at that speed is equally likely to fall on either side of the criterion. Confidence intervals on a crossover speed were calculated from a 500-replicate cluster bootstrap that resamples the 13 individuals because steps within a bird are not independent.

Every table cell presents the median (interquartile range) unless otherwise specified. Each measure is rounded to the number of decimal places needed to show the narrowest interquartile width to two significant figures, taken over the steady, accelerating and decelerating cells together so that Table 1 and Table S4 share one format. Continuous descriptors (recovery, congruity, collision angle, duty factor, step length and step frequency) are displayed with penalized-spline trends fitted to equal-width speed-bin means, so the sparse speed extremes are not overweighted by the dense mid-speed range.

Agreement between two binary labels is summarized three ways: the raw percent agreement, Cohen's *κ* and the *φ* coefficient, and the Pearson correlation between the labels. The interval on the percent agreement comes from a cluster bootstrap over individuals, as was done for the crossover speeds, because steps within a bird are not independent. All three are reported because counterclockwise steps are only 16% of the sample, which inflates raw agreement relative to the chance-corrected measures.

Mean traces and mean loops are group means with Student-*t* 95% confidence bands, formed over steps and over complete strides, respectively, with each observation assigned to its gait and speed bin from its own values. Step-cycle traces are averaged at each percentage of the cycle, and the time axis is scaled by the group's mean cycle duration. Stride loops are averaged after registering each stride to a continuous phase taken from the analytic signal (Hilbert transform) of the vertical CoM velocity. Phase advances continuously across the step join, so the velocity extremes align before averaging.

### Software used

Signal processing, CoM reconstruction and per-step measures were computed in MATLAB (R2019b or later; MathWorks, Natick, MA, USA) and the statistics and figures in R 4.6.0, using the packages mgcv, cluster, dplyr and ggplot2.

### Use of AI tools

Claude Code was used with careful human review to improve analysis code, automate the analysis pipeline, collate results into tables, generate data visualizations, and edit the text to clarify language. All AI-assisted outputs were carefully reviewed by the authors.

## Supplementary Material



10.1242/biolopen.062880_sup1 Supplementary information
