# Supplementary material for: A velocity-loop view of avian gait: hodograph rotation sense characterizes the walking-to-running continuum in guinea fowl
Source: Biol Open. 2026 Sep 18;15(9):bio062880. doi: 10.1242/bio.062880 (PMC13617982; doi:10.1242/bio.062880)
Supplement: Supplementary file 1 [file biolopen-15-062880-s1.pdf]

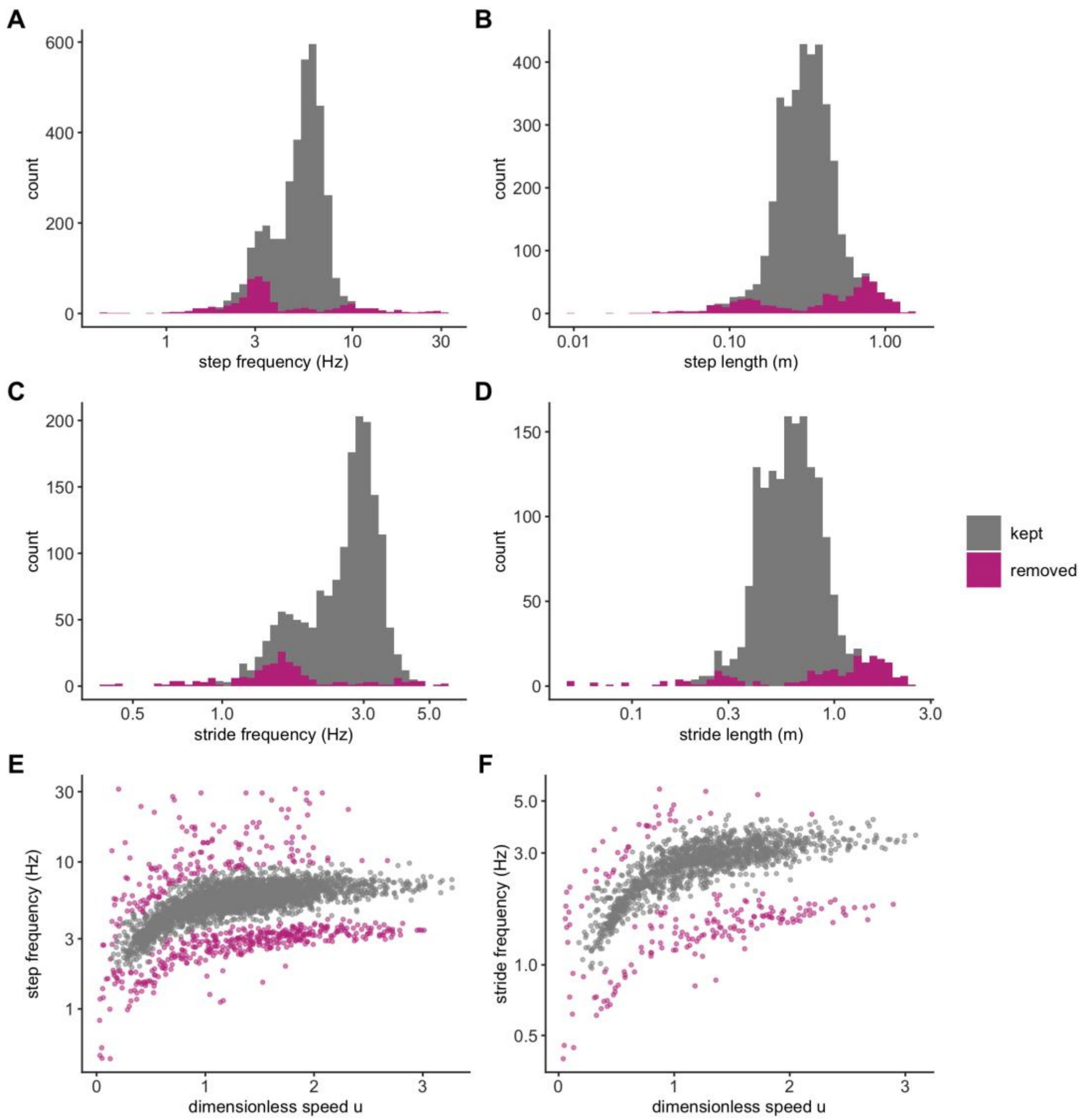

Fig. S1. Outlier-filter visualization. Step and stride frequency and length distributions with the log-MAD keep boundaries (k = 3.0) and the flagged observations. A value is flagged if it exceeds the boundary on frequency or on length, or if the speed-conditional filter flags it as a merged (half-frequency) or split (double-frequency) mis-cut cycle whose frequency departs from the speed-local median. The filters removed 584 of 3842 steps and 205 of 1678 strides.

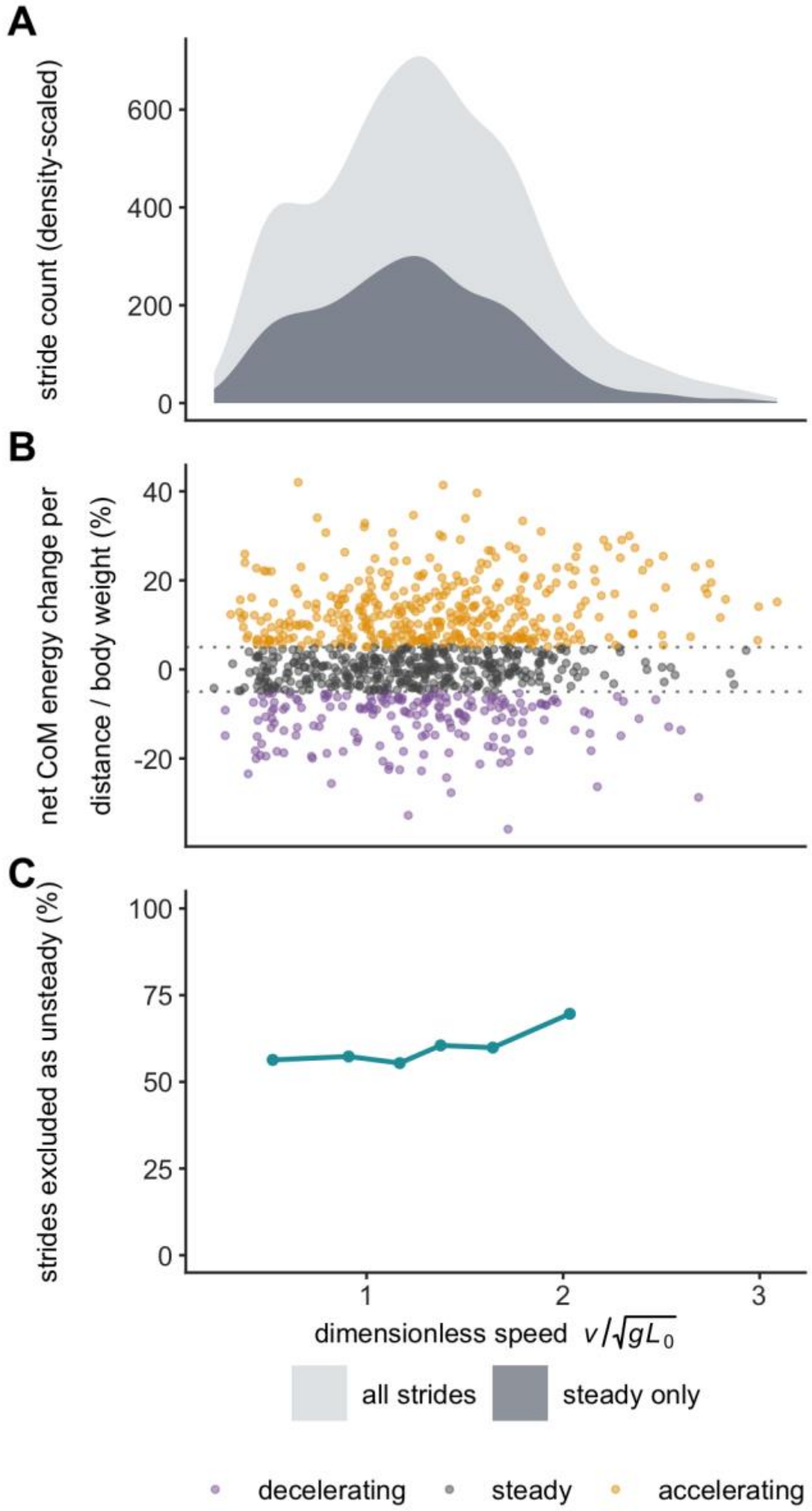

Fig. S2. The steadiness criterion excludes a similar fraction of strides at every speed. (A) Speed coverage of the steady subset against the full sample. (B) Energy grade against speed with the ±5% band, colored by steadiness class. (C) Fraction of strides excluded as unsteady, in six equal-count speed bins.

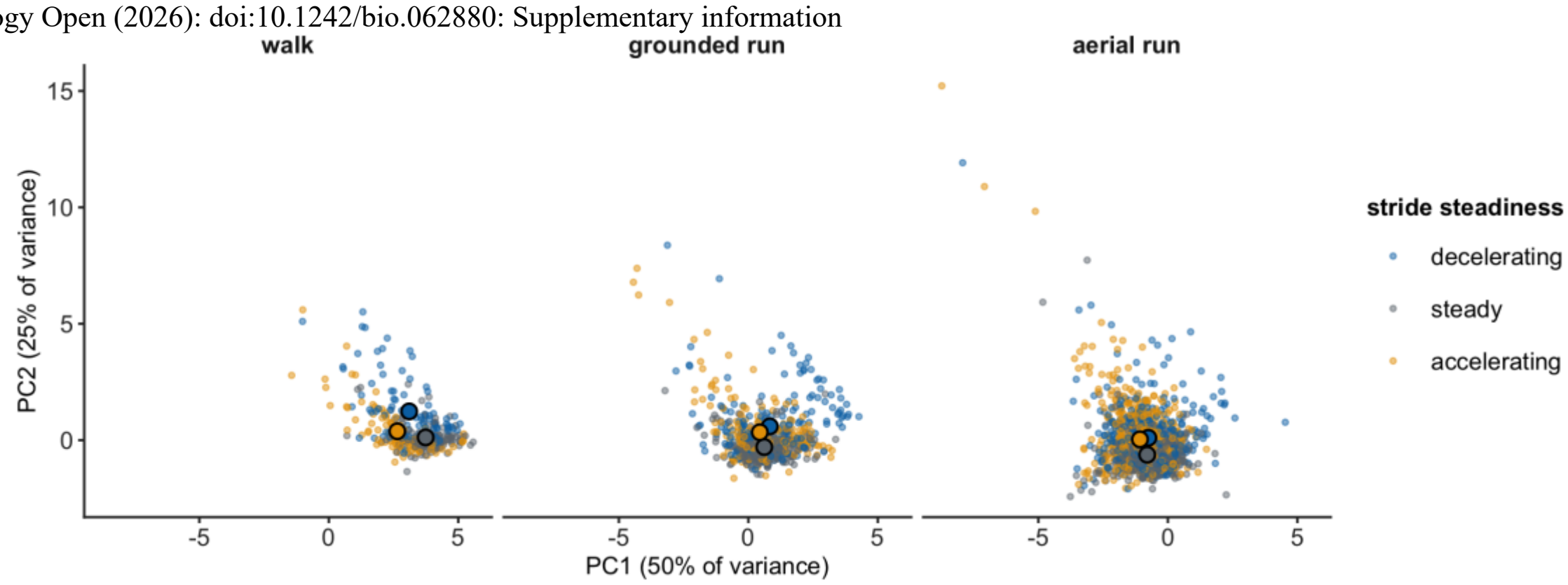

Fig. S3. Steadiness orders the centroids along the second principal component. PC1 against PC2 ($n = 2580$ steps), colored by stride steadiness; large symbols are the per-gait, per-steadiness centroids.

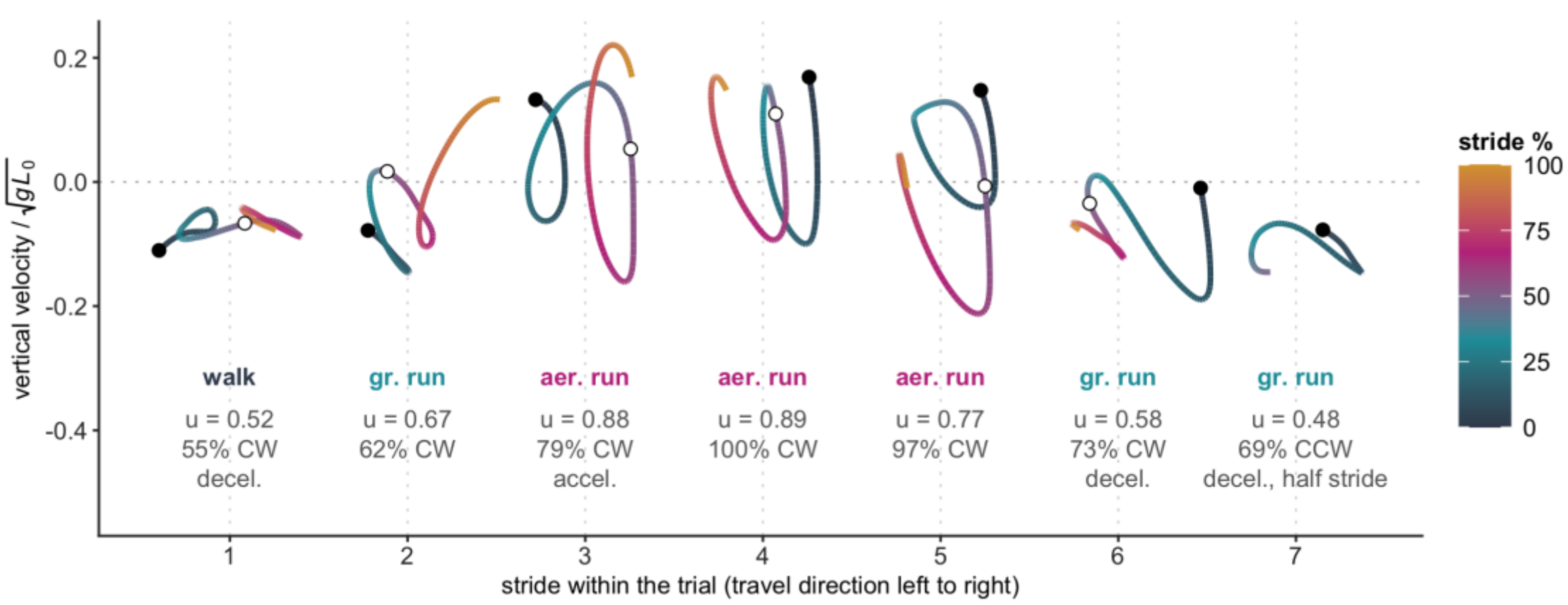

Fig. S4. Within a single trial, the velocity loop grows and shrinks with speed. Every stride of one trial (bird rvc_yel, six full strides and a trailing half stride), drawn as its raw sampled velocity trajectory: fore-aft velocity fluctuation about the stride mean against vertical velocity, both normalized by $\sqrt{gL_0}$. Color is percent of stride, the filled dot marks the stride touchdown and the open dot the contralateral touchdown. Loops are laid out in travel order, which follows the trial's rise-and-fall speed arc from $u = 0.52$ to 0.89 and back to 0.48. The percentage is the larger share of the loop's enclosed area, and its sense is the stride's rotation sense.

Biology Open • Supplementary information

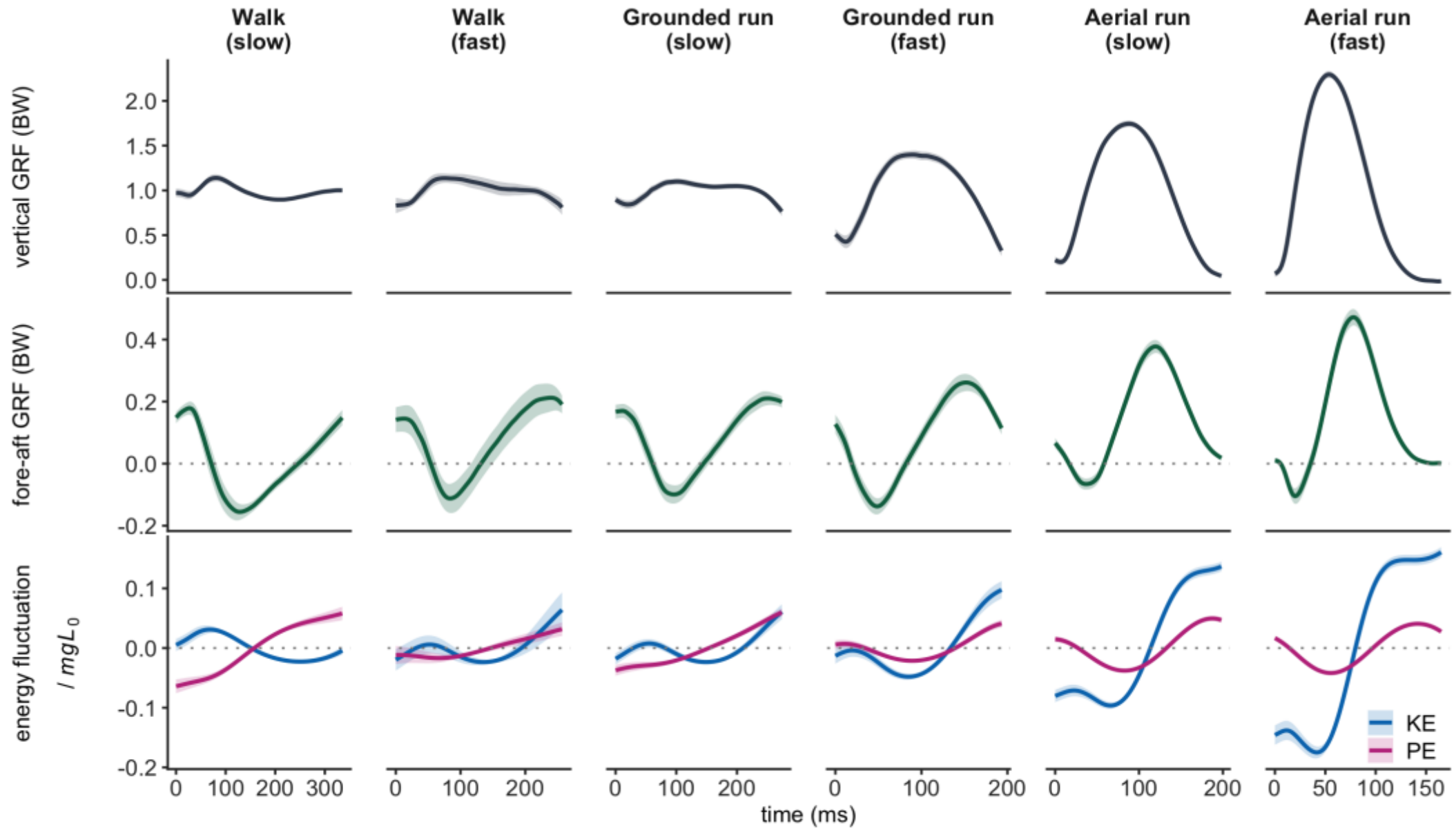

	Walk (slow)	Walk (fast)	Grounded run (slow)	Grounded run (fast)	Aerial run (slow)	Aerial run (fast)
Fr	0.16 [0.04 0.25]	0.44 [0.25 1.25]	0.42 [0.04 0.76]	1.11 [0.77 2.11]	1.53 [0.06 2.31]	3.97 [2.32 10.69]
u	0.39 [0.21 0.50]	0.64 [0.50 1.12]	0.62 [0.21 0.87]	1.05 [0.87 1.45]	1.22 [0.24 1.52]	1.96 [1.52 3.27]
v (m/s)	0.54 [0.28 0.71]	0.90 [0.70 1.64]	0.87 [0.30 1.29]	1.48 [1.22 2.02]	1.74 [0.34 2.24]	2.79 [2.15 4.67]
n steps	60	45	122	79	318	323

Fig. S5. Accelerating steps show the same pendular-to-bouncing progression as steady steps. Group mean step-cycle traces for accelerating steps with 95% confidence bands, each gait split into slow and fast at its median speed. Rows as in Fig. 7.

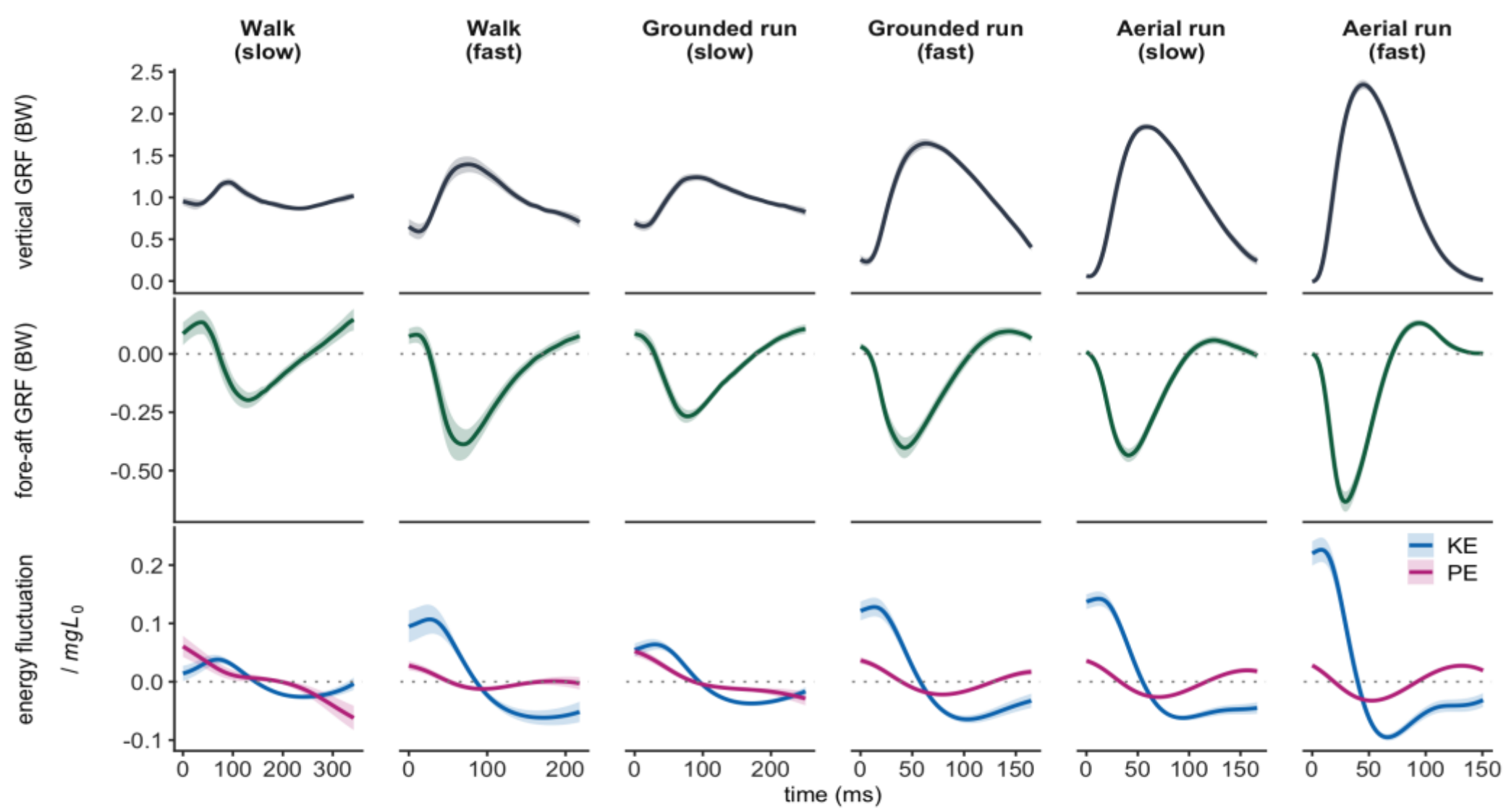

	Walk (slow)	Walk (fast)	Grounded run (slow)	Grounded run (fast)	Aerial run (slow)	Aerial run (fast)
Fr	0.18 [0.02 0.25]	0.69 [0.26 2.87]	0.42 [0.02 0.76]	1.37 [0.77 4.34]	1.60 [0.04 2.31]	3.64 [2.32 9.02]
u	0.41 [0.14 0.50]	0.78 [0.51 1.69]	0.62 [0.16 0.87]	1.15 [0.88 2.08]	1.25 [0.19 1.52]	1.88 [1.52 3.00]
v (m/s)	0.57 [0.20 0.69]	1.10 [0.70 2.37]	0.87 [0.22 1.28]	1.63 [1.22 2.94]	1.78 [0.27 2.26]	2.68 [2.11 4.29]
n steps	33	64	93	111	195	207

Fig. S6. Decelerating steps show the same pendular-to-bouncing progression as steady steps. Group mean step-cycle traces for decelerating steps with 95% confidence bands, each gait split into slow and fast at its median speed. Rows as in Fig. 7.

Table S1. Principal component analysis of the six center-of-mass-dynamics descriptors ($n = 2580$ steps). Loadings of all six components and the variance explained.

Descriptor	PC1	PC2	PC3	PC4	PC5	PC6
Pendular recovery	0.401	0.359	0.448	0.560	-0.443	0.017
KE-PE congruity	-0.414	-0.230	-0.522	0.665	-0.247	0.021
Mechanical cost of transport	-0.374	0.608	-0.020	0.042	0.160	-0.680
Collision angle	-0.407	0.562	-0.017	-0.111	-0.043	0.710
Touchdown energy	-0.380	-0.267	0.623	0.349	0.515	0.094
Duty factor	0.467	0.248	-0.371	0.329	0.671	0.155
Variance explained (%)	50.0	24.7	15.9	5.2	3.3	1.0
Cumulative variance (%)	50.0	74.7	90.5	95.7	99.0	100.0

Table S2. Pearson correlations among the six center-of-mass-dynamics descriptors and the hodograph signed area ($n = 2580$ steps). Columns are numbered to the rows. The signed area is appended to the matrix and is not an input to the principal component analysis or the clustering.

Descriptor	1	2	3	4	5	6	7
1 Pendular recovery	1.000	-0.705	-0.143	-0.214	-0.318	0.534	0.453
2 KE-PE congruity	-0.705	1.000	0.267	0.303	0.299	-0.444	-0.333
3 Mechanical cost of transport	-0.143	0.267	1.000	0.931	0.191	-0.274	-0.480
4 Collision angle	-0.214	0.303	0.931	1.000	0.220	-0.368	-0.569
5 Touchdown energy	-0.318	0.299	0.191	0.220	1.000	-0.746	-0.365
6 Duty factor	0.534	-0.444	-0.274	-0.368	-0.746	1.000	0.529
7 Hodograph signed area	0.453	-0.333	-0.480	-0.569	-0.365	0.529	1.000

Table S3. Cluster structure of the six center-of-mass-dynamics descriptors and agreement of the two-cluster partition with hodograph rotation sense ($n = 2580$ steps). Panel C uses the three-gait form, in which every step with a flight phase counts as aerial running, so its aerial-run count exceeds the four-cell count in Table 1. (A) Average silhouette width and cluster sizes for $k = 2$ to 6. (B) The $k = 2$ partition against rotation sense, overall and (C) within each gait.

(A) Cluster tendency

Clusters k	Average silhouette width	Cluster sizes
2	0.421	1997 / 583
3	0.253	1045 / 1014 / 521
4	0.267	922 / 710 / 495 / 453

Biology Open • Supplementary information

Clusters k	Average silhouette width	Cluster sizes
5	0.188	657 / 566 / 484 / 470 / 403
6	0.177	654 / 502 / 470 / 397 / 303 / 254

(B) The $k = 2$ partition against rotation sense

Quantity	Value
Steps compared	2580
Both pendular (pendular cluster, pendular rotation sense)	343
Cluster pendular, rotation sense bouncing	240
Cluster bouncing, rotation sense pendular	78
Both bouncing (bouncing cluster, bouncing rotation sense)	1919
Percent agreement	87.7
Percent agreement, cluster bootstrap 95% CI lower	84.0
Percent agreement, cluster bootstrap 95% CI upper	91.9
Cohen's κ	0.609
Cohen's κ, 95% CI half-width	0.038
ϕ coefficient (Pearson r of the two binary labels)	0.622
Chance agreement expected under independence	0.685

(C) Agreement within each gait

Gait	Steps	Agreement (%)
Walk	329	80.2
Grounded run	634	74.1
Aerial run	1617	94.5

Table S4. Center-of-mass dynamics of unsteady steps, by gait cell. Values as in Table 1.

(A) Accelerating steps

Measure	Walk (grounded)	Grounded run	Aerial run	Pendular run
# steps of all	105 of 329	201 of 634	598 of 1544	43 of 73
Dimensionless speed, u	0.46 (0.38 to 0.56)	0.79 (0.60 to 0.99)	1.52 (1.26 to 1.85)	1.63 (1.13 to 1.95)
Froude number, u^2	0.21 (0.14 to 0.31)	0.62 (0.37 to 0.97)	2.31 (1.58 to 3.41)	2.65 (1.29 to 3.80)
PC1 score	2.71 (2.06 to 3.36)	0.42 (-0.26 to 1.36)	-1.03 (-1.53 to -0.58)	-0.43 (-1.29 to 0.25)
PC2 score	0.02 (-0.23 to 0.71)	-0.00 (-0.36 to 0.52)	-0.31 (-0.76 to 0.36)	0.93 (0.09 to 1.65)
Hodograph rotation sense (% CCW / % CW)	85 / 15	20 / 80	1 / 99	14 / 86
Hodograph signed area, $A/(gL_0)$	0.0058 (0.0016 to 0.0087)	-0.0084 (-0.0154 to -0.0017)	-0.0423 (-0.0615 to -0.0272)	-0.0459 (-0.0629 to -0.0125)
Pendular recovery (%)	29.8 (20.4 to 36.2)	5.0 (1.2 to 12.8)	2.6 (1.0 to 5.6)	12.9 (8.6 to 18.8)
KE-PE congruity (%)	41.5 (31.3 to 46.2)	73.6 (59.4 to 86.1)	75.9 (66.1 to 82.7)	45.9 (42.7 to 47.9)
Mechanical cost of transport	0.152 (0.119 to 0.213)	0.220 (0.186 to 0.259)	0.251 (0.221 to 0.288)	0.306 (0.223 to 0.343)
Collision angle (deg)	8.8 (6.7 to 11.5)	12.2 (11.0 to 14.6)	14.8 (13.1 to 16.9)	17.3 (14.1 to 20.4)
Touchdown mechanical energy, $E/(mgL_0)$	0.91 (0.79 to 1.06)	1.09 (0.92 to 1.29)	1.95 (1.62 to 2.53)	2.06 (1.54 to 2.53)
Leg stiffness,	7.2 (4.9 to 9.5)	6.2 (5.3 to 8.2)	10.6 (8.2 to 13.3)	16.9 (12.1 to 29.7)

Biology Open • Supplementary information

Measure	Walk (grounded)	Grounded run	Aerial run	Pendular run
$kL_0/(mg)$				
Steps with a leg-stiffness fit	93	195	595	43
Duty factor	0.672 (0.645 to 0.708)	0.583 (0.509 to 0.647)	0.379 (0.334 to 0.430)	0.343 (0.312 to 0.436)

(B) Decelerating steps

Measure	Walk (grounded)	Grounded run	Aerial run	Pendular run
# steps of all	97 of 329	204 of 634	378 of 1544	24 of 73
Dimensionless speed, u	0.57 (0.47 to 0.80)	0.92 (0.71 to 1.13)	1.55 (1.30 to 1.82)	1.24 (1.11 to 1.57)
Froude number, u^2	0.33 (0.23 to 0.64)	0.84 (0.50 to 1.27)	2.41 (1.69 to 3.32)	1.55 (1.23 to 2.46)
PC1 score	3.34 (2.34 to 4.01)	0.64 (-0.10 to 1.74)	-0.79 (-1.41 to -0.19)	1.30 (0.20 to 2.05)
PC2 score	0.73 (0.26 to 1.73)	0.21 (-0.35 to 1.12)	-0.22 (-0.72 to 0.43)	1.16 (0.60 to 2.24)
Hodograph rotation sense (% CCW / % CW)	66 / 34	18 / 82	11 / 89	46 / 54
Hodograph signed area, $A/(gL_0)$	0.0037 (-0.0030 to 0.0081)	-0.0124 (-0.0194 to -0.0040)	-0.0156 (-0.0235 to -0.0070)	-0.0084 (-0.0224 to 0.0080)
Pendular recovery (%)	73.4 (59.3 to 92.3)	16.6 (6.9 to 46.9)	7.9 (2.7 to 18.9)	64.4 (41.2 to 88.7)
KE-PE congruity (%)	39.0 (31.8 to 45.2)	69.5 (57.8 to 80.5)	77.3 (69.5 to 83.6)	42.7 (40.9 to 45.0)
Mechanical cost of transport	0.159 (0.123 to 0.239)	0.222 (0.191 to 0.271)	0.259 (0.221 to 0.305)	0.247 (0.223 to 0.279)
Collision angle (deg)	9.3 (7.2 to 13.4)	12.4 (10.9 to 14.6)	14.2 (12.5 to 15.9)	13.9 (13.0 to 15.6)
Touchdown mechanical energy, $E/(mgL_0)$	1.12 (0.95 to 1.28)	1.35 (1.17 to 1.59)	2.24 (1.86 to 2.71)	1.96 (1.55 to 2.41)
Leg stiffness, $kL_0/(mg)$	6.4 (5.0 to 10.5)	6.5 (5.5 to 8.9)	9.2 (7.8 to 11.2)	7.0 (6.0 to 8.7)
Steps with a leg-stiffness fit	82	187	360	22
Duty factor	0.666 (0.596 to 0.694)	0.564 (0.499 to 0.637)	0.423 (0.368 to 0.477)	0.462 (0.421 to 0.551)

Table S5. Morphology and sample size for each bird-session. Body mass and leg length L_0 per bird-session, with the source of L_0: the session's own non-aerial steps, that bird's non-aerial steps pooled over its sessions, or the within-sample allometry.

Session	Collection	Session date	Mass (kg)	L_0 (m)	L_0 source	Steps	Strides
pot_bla	Drop versus pothole	2012-02	1.158	0.208	session	169	77
pot_blu	Drop versus pothole	2012-02	1.087	0.205	session	133	60
pot_g00	Drop versus pothole	2012-02	1.313	0.207	session	119	52
pot_gry	Drop versus pothole	2012-02	1.377	0.222	session	140	61
pot_reb	Drop versus pothole	2012-02	1.177	0.219	session	134	60
pot_red	Drop versus	2012-02	1.243	0.204	session	181	83

Biology Open • Supplementary information

Session	Collection	Session date	Mass (kg)	L_0 (m)	L_0 source	Steps	Strides
	pothole						
pot_rre	Drop versus pothole	2012-02	1.253	0.208	allometry	52	20
pot_yel	Drop versus pothole	2012-02	1.172	0.198	session	121	53
surf_bla	Surface variation	2012-06	1.453	0.222	session	190	87
surf_g00	Surface variation	2012-06	1.740	0.223	session	116	52
surf_gry	Surface variation	2012-06	1.492	0.232	session	111	48
surf_noc	Surface variation	2012-06	1.361	0.210	session	117	52
surf_red	Surface variation	2012-06	1.332	0.214	session	129	58
surf_rre	Surface variation	2012-06	1.424	0.208	allometry	68	26
surf_yel	Surface variation	2012-06	1.302	0.227	session	93	39
rvc_blu	Level and obstacle	2008-11	1.161	0.203	session	18	8
rvc_blu	Level and obstacle	2009-05	1.378	0.200	session	113	48
rvc_blu	Level and obstacle	2009-11	1.286	0.203	bird	42	14
rvc_bly	Level and obstacle	2008-11	1.277	0.197	bird	14	4
rvc_bly	Level and obstacle	2009-05	1.653	0.197	session	471	214
rvc_bly	Level and obstacle	2009-11	1.273	0.197	bird	80	32
rvc_red	Level and obstacle	2008-11	1.380	0.216	session	29	12
rvc_red	Level and obstacle	2009-05	1.934	0.197	session	286	128
rvc_red	Level and obstacle	2009-11	1.338	0.200	bird	77	30
rvc_rey	Level and obstacle	2008-11	1.317	0.193	session	9	4
rvc_rey	Level and obstacle	2009-05	1.771	0.192	session	363	161
rvc_rey	Level and obstacle	2009-11	1.276	0.208	session	107	44
rvc_yel	Level and obstacle	2008-11	1.203	0.195	session	31	13
rvc_yel	Level and obstacle	2009-04	1.504	0.197	session	284	122
rvc_yel	Level and obstacle	2009-11	1.190	0.197	bird	45	16

Biology Open • Supplementary information
