# Supplementary material for: A velocity-loop view of avian gait: hodograph rotation sense characterizes the walking-to-running continuum in guinea fowl
Source: Biol Open. 2026 Sep 18;15(9):bio062880. doi: 10.1242/bio.062880 (PMC13617982; doi:10.1242/bio.062880)
Supplement: Supplementary file 2 [file biolopen-15-062880_review_history.pdf]

A velocity-loop view of avian gait: hodograph rotation sense characterizes the walking-to-running continuum in guinea fowl

Aleksandra Birn-Jeffery and Monica A. Daley
10.1242/bio.062880

Editor: Valentina di Santo

Review timeline

Original submission: 17 August 2026
Accepted: 25 August 2026

Original submission

First decision letter

MS ID#: bio.062880

MS Title: A velocity-loop view of avian gait: hodograph rotation sense characterizes the walking-to-running continuum in guinea fowl

Authors: Monica A. Daley; Aleksandra Birn-Jeffery

I am happy to tell you that your manuscript has been accepted for publication in Biology Open, pending our standard publication integrity checks. It was accepted on 25th August 2026.

Reviewer 1

Comments for the author

Overall, I enjoyed reading this manuscript. The study addresses an interesting and important question regarding gait identification in birds and presents a novel application of hodograph analysis. The work appears to have been carefully conducted, the manuscript is clearly written, the rationale and methods and interpretations are easy to follow. I have only a few minor comments and points of clarification for the authors to consider.

Birds utilise walking, grounded running and aerial running as speed increases. Unlike humans, where gaits are discrete and characterised by distinct mechanical and kinematic features, gait transitions in birds occur along a continuum, with these parameters changing continuously with speed. Since footfall pattern and duty factor alone are insufficient to classify gait, Daley and Birn-Jeffrey sought to determine whether hodographs (CoM velocity loops) could be used to identify the transitions between gaits in ground-dwelling birds. The rationale is that the direction of loop rotation depends on whether KE and PE fluctuations are in or out of phase. The authors therefore predicted that loop rotation direction would change as birds transition from walking to grounded running.

The study provides a validated approach for identifying gait dynamics from CoM fluctuations, which can be derived from either force plates (as in the present study) or accelerometry. The method may offer advantages over traditional measures such as pendular energy recovery and KE-PE congruity.

© 2026. Published by The Company of Biologists under the terms of the Creative Commons Attribution License (https://creativecommons.org/licenses/by/4.0/).

The introduction clearly sets out the problem the study aims to address and provides appropriate detail with reference to the relevant literature. However, it would be helpful to clarify exactly what the velocity loop represents (i.e. a plot of vertical velocity vs. horizontal velocity).

The methods are detailed and clearly described. However, the estimated position of the CoM appears to be defined differently in two sections of the manuscript. At L416, the CoM is described as the mean of the cranial and caudal back markers, whereas at L470-471 it is described as an offset position relative to the mean of the back markers. This discrepancy should be clarified. In addition, I do not believe that CoT is defined (L557).

Fig. 5. Why in "walk, fast" does the hodograph loop not close at the end of the stride? A small offset, as seen for the other gaits would be expected, but the offset for this condition appears considerably larger.

Fig. 7. Consider moving the "KE, PE" label closer to the corresponding energy fluctuation traces to improve clarity.

The authors report that hodograph rotation agreed with mechanical gait descriptors in 88% of steps, with close agreement between hodograph-derived and mechanically derived transition speeds. This finding supports the use of hodograph rotation as a robust indicator of gait dynamics.

<u>Reviewer 2</u>

Comments for the author

This manuscript studies terrestrial gaits and their transitions in birds by focusing on a large dataset of guinea fowl terrestrial locomotion across a large range of speeds. This dataset very interestingly shows that gaits in guinea fowl are not discrete (as in humans) but instead lie along a continuum from pendular to bouncing center-of-mass. The authors go on to use information from hodographs of the motion of the center-of-mass to show that the rotation and size of the hodograph for a step is a variable that allows for distinguishing pendulum-like walking from bouncy grounded+aerial running.

The overall experimental and methodological quality of this manuscript seem high and the dataset and methods seem highly appropriate for addressing the author's proposed questions. The large number of strides or steps in the dataset across a wide range of speeds is commendable and inspires strong confidence in the results. The methods section also provides an excellent level of detail in how data were collected and then filtered to produce this dataset. The author's conclusions seem well-supported by the data and the figures, statistics, and citations all seem of sufficient and high quality.

I have very few (and rather paltry) comments concerning this excellent manuscript. My two general ones will be followed by three in-line comments that are mostly about clarifying language in a few places.

1.The main results of this manuscript are that guinea fowl terrestrial locomotion is best described as a continuum, but at the same time you ARE able to make some distinctions of gaits along this continuum using the information in the hodographs. I may be oversimplifying these results, but at some level there is a slight contradiction here - basically, how can one distinguish gaits along a continuum? I think it would be worth being a bit more explicit in your discussion about this sort of question. Perhaps you could offer readers a few sentences of explanation that captures these sorts of thoughts.

2. This paper is well-written and well-designed, but it will be difficult to understand for anyone not well-versed in terrestrial gait biomechanics. I think this audience is definitely the ones who will be reading this very cool work, but the authors could also spend space briefly defining more of the various specialized terms if they wanted to make this work more easily readable by non-experts. Obviously this is not necessary to improve the paper and will come at a cost of making the paper longer - I leave this decision fully up to the authors.

© 2026. Published by The Company of Biologists under the terms of the Creative Commons Attribution License (https://creativecommons.org/licenses/by/4.0/).

Line 47: "CoM" - I may have missed it but I don't see the abbreviation for center of mass was introduced yet. I think this is the first time it's used - please introduce the abbreviation here.

Line 65: "whereas in humans all but one changed". I think what is meant here is "in humans, all but one kinematic/kinetic parameter changed", but I'm not totally sure - could also be "all but one individual" or even all but one population of humans or some other category. Could you just add a work or two here to clarify?

Line 481-482: "By cohort the 2012 birds, which are the collections that report supplied,[…]". Confusing phrasing - unsure of meaning. Please revise for clarity.

Reviewer's Responses to Questions

Experimental quality
Are experiments performed using appropriate methods that will answer the question (or test the hypothesis or support the observations) posed by the authors? Is the right tool used for the job?
If 'No', please indicate reasons in Comments for Author box below.
Reviewer #1:

- Yes

Reviewer #2:

- Yes

Were the data analyzed using appropriate statistical tests?
If 'No', please indicate reasons in Comments for Author box below.
Reviewer #1:

- Yes

Reviewer #2:

- Yes

Reproducibility
Were experiments performed using adequate number of biological replicates?
If 'No', please indicate reasons in Comments for Author box below.
Reviewer #1:

- Yes

Reviewer #2:

- Yes

Is there sufficient raw data to assess the rigor of the analysis?
If 'No', please indicate reasons in Comments for Author box below.
Reviewer #1:

- Yes

Reviewer #2:

- Yes

Does the methods section provide sufficient detail to permit reproducibility?
If 'No', please indicate reasons in Comments for Author box below.
Reviewer #1:

- Yes

Reviewer #2:

- Yes

Completeness
Are the manuscript's conclusions supported by the data?
If 'No', please indicate reasons in Comments for Author box below.
Reviewer #1:

- Yes

Reviewer #2:

© 2026. Published by The Company of Biologists under the terms of the Creative Commons Attribution License (https://creativecommons.org/licenses/by/4.0/).

- Yes

Scholarship
Do the authors cite and discuss the merits of data that would argue for and against their conclusion?
If 'No', please indicate reasons in Comments for Author box below.
Reviewer #1:
- Yes

Reviewer #2:
- Yes

Does the manuscript title & abstract accurately reflect the contents of the manuscript, without hyperbole?
If 'No', please indicate reasons in Comments for Author box below.
Reviewer #1:
- Yes

Reviewer #2:
- Yes

© 2026. Published by The Company of Biologists under the terms of the Creative Commons Attribution License (https://creativecommons.org/licenses/by/4.0/).
